# Prostaglandin EP2 receptor antagonist ameliorates neuroinflammation in a two-hit mouse model of Alzheimer’s disease

**DOI:** 10.1186/s12974-021-02297-7

**Published:** 2021-11-20

**Authors:** Avijit Banik, Radhika Amaradhi, Daniel Lee, Michael Sau, Wenyi Wang, Raymond Dingledine, Thota Ganesh

**Affiliations:** Department of Pharmacology and Chemical Biology, School of Medicine, Emory University, Atlanta, GA 30322 USA

**Keywords:** Two-hit 5xFAD, EP2, Lipopolysaccharide, TG11-77.HCl, Neuroinflammation, Microgliosis

## Abstract

**Background:**

Alzheimer’s disease (AD) causes substantial medical and societal burden with no therapies ameliorating cognitive deficits. Centralized pathologies involving amyloids, neurofibrillary tangles, and neuroinflammatory pathways are being investigated to identify disease-modifying targets for AD. Cyclooxygenase-2 (COX-2) is one of the potential neuroinflammatory agents involved in AD progression. However, chronic use of COX-2 inhibitors in patients produced adverse cardiovascular effects. We asked whether inhibition of EP2 receptors, downstream of the COX-2 signaling pathway, can ameliorate neuroinflammation in AD brains in presence or absence of a secondary inflammatory stimuli.

**Methods:**

We treated 5xFAD mice and their non-transgenic (nTg) littermates in presence or absence of lipopolysaccharide (LPS) with an EP2 antagonist (TG11-77.HCl). In cohort 1, nTg (no-hit) or 5xFAD (single-hit—genetic) mice were treated with vehicle or TG11-77.HCl for 12 weeks. In cohort 2, nTg (single-hit—environmental) and 5xFAD mice (two-hit) were administered LPS (0.5 mg/kg/week) and treated with vehicle or TG11-77.HCl for 8 weeks.

**Results:**

Complete blood count analysis showed that LPS induced anemia of inflammation in both groups in cohort 2. There was no adverse effect of LPS or EP2 antagonist on body weight throughout the treatment. In the neocortex isolated from the two-hit cohort of females, but not males, the elevated mRNA levels of proinflammatory mediators (IL-1β, TNF, IL-6, CCL2, EP2), glial markers (IBA1, GFAP, CD11b, S110B), and glial proteins were significantly reduced by EP2 antagonist treatment. Intriguingly, the EP2 antagonist had no effect on either of the single-hit cohorts. There was a modest increase in amyloid–plaque deposition upon EP2 antagonist treatment in the two-hit female brains, but not in the single-hit genetic female cohort.

**Conclusion:**

These results reveal a potential neuroinflammatory role for EP2 in the two-hit 5xFAD mouse model. A selective EP2 antagonist reduces inflammation only in female AD mice subjected to a second inflammatory insult.

**Supplementary Information:**

The online version contains supplementary material available at 10.1186/s12974-021-02297-7.

## Introduction

Alzheimer's disease (AD) is a neurodegenerative disorder that is the leading cause of dementia in the elderly [1]. AD leads to progressive loss of cognitive functions including memory, judgment, and ability to reach a decision [2]. The accumulation of extracellular amyloid-β (Aβ) plaques and intracellular neurofibrillary tangles (NFTs) in the brain are the pathological hallmarks of AD. However, human subjects who show accumulated Aβ can be presymptomatic, suggesting Aβ accumulation is necessary but not sufficient for the development of full spectrum of AD [3, 4]. The last two decades of drug discovery efforts for AD have mainly focused on the amyloid cascade hypothesis [5, 6], which involves targeting the removal of amyloid plaques from the brain of AD patients with active or passive immunization (e.g., bapineuzumab), decreasing total Aβ production with γ-secretase inhibitors (e.g., semagacestat), or decreasing Aβ42 levels with γ-secretase modulators (e.g., tarenflurbil). However, these agents have not yet provided a clinical benefit [7–9] suggesting anti-amyloid therapeutics alone may not be sufficient to ameliorate the disease pathology. The currently approved therapeutics (inhibitors of cholinesterase or a NMDA receptor antagonist) offer minor symptomatic improvement and little, if any, modification of disease progression [10]. Thus, identification of novel biological targets, and drugs that work through novel mechanisms of action are crucial goals for future AD therapies [11–13].

Neuroinflammation associated with AD was initially assumed to be a consequence of the pathological events, hence underappreciated as a therapeutic target [14–16]. However, recent evidence established that neuroinflammation exacerbates AD pathology [17, 18] suggesting it could be a target for drug discovery. Moreover, it has been shown that neuroinflammation with neurofibrillary tangles [19] or neurodegeneration-related biomarkers [20] show a stronger correlation with cognitive decline compared to Aβ-accumulation alone in AD patients. However, the underlying sequence of events that leads to the clinical symptoms is yet to be understood. Furthermore, the AD brain having a multifactorial etiology, neurodegeneration alone is not pinned to the disease pathology. Instead, multiple environmental stimuli, such as infection, poor diet, smoking, metal toxicity and other associated comorbidities in early life, termed as “two-hit” manifestations, are suggested to play an intrinsic role in accelerating AD pathologies at later stages [21, 22]. Experimentally, lipopolysaccharide (LPS) can be added to a genetic AD model to replicate such “two-hit” models to demonstrate the associative links between neurodegeneration and neuroinflammation in many neurodegenerative disorders [23]. LPS is known to cause neuroinflammation by activating inflammasomes in the brain or through peripheral inflammation and breakdown of blood brain barrier [24, 25].

Neuroinflammation is a broad term widely used to indicate activation of microglia and astrocytes, upregulation of pro-inflammatory cytokines and chemokines, and induction of inflammatory mediators including cyclooxygenase-2 (COX-2) [26, 27]. These neuroinflammatory markers were found in patients during the progression of AD [28]. For example, increased COX-2 levels were found in the early stage of AD brain and its levels were correlated with levels of Aβ-peptide [29]. Elevated COX-2 levels are also correlated with neuronal atrophy and Aβ-plaque density in hippocampal neuronal structures [30, 31]. The highest expression of COX-2 was found at the presymptomatic stage of AD according to Braak stages [32]. A non-selective COX-2 inhibitor (naproxen) reduced the incidence of AD when it was given to patients before the phase of subjective cognitive impairment, but it failed to benefit clinically diagnosed patients [33], reinforcing the notion that the presymptomatic stage of AD offers therapeutic opportunities [3, 4].

COX-2 catalyzes the synthesis of five prostaglandins (PGD_2_, PGE_2_, PGF_2_, PGI_2_ and TXA_2_), which activate nine prostanoid receptors. These receptors play a physiological and or harmful pathological role in a variety of conditions, such as cancers, cardiovascular and neurodegenerative disorders [34–36]. For example, the IP receptor activated by PGI_2_ plays a cardioprotective role [37, 38]. The adverse cardiac effects of COX-2 inhibitors are mediated mainly by suppression of IP receptor activation [39–41]. Thus, targeting a specific pro-inflammatory prostanoid receptor could be a superior therapeutic strategy, compared to generic inhibition of the entire COX-2 signaling cascade, for the suppression of AD related pathologies [42].

The EP2 receptor, which is downstream in the COX-2 signaling pathway and activated by prostaglandin-E_2_ (PGE_2_), is expressed in brain regions including hippocampus and cerebral cortex that are significantly affected in AD [43]. It has been shown that microglia lacking EP2 displayed stronger Aβ-phagocytosis, and reduced Aβ-induced neurotoxicity [44]. Moreover, global deletion of EP2 in the APP-PS1 mouse model of AD resulted in significant reduction of oxidative stress and levels of Aβ_40_ and Aβ_42_ peptides [45]. Conditional deletion of EP2 in microglia reduced spatial memory deficits in the APP-PS1 model, indicating that EP2 activation attenuates beneficial functions of microglia in AD models [46]. Moreover, a selective PGE_2_ receptor EP2 antagonist also enhances Aβ-phagocytosis in vitro in a macrophage cell line [47]. Furthermore, our recent in vitro study revealed that in an activated state, a murine microglial cell line stably transfected with human EP2 receptors (BV2-hEP2), when challenged with a specific EP2 receptor agonist, produced inflammation that was competitively inhibited by a selective EP2 antagonist [48, 49]. With these rationales, here we investigated whether a selective and potent EP2 antagonist, ameliorates neuroinflammation in 5xFAD model in the presence (two-hit), and absence (single-hit—genetic) of a second peripheral inflammatory insult by LPS. We report that chronic EP2 antagonism is beneficial only in the two-hit 5xFAD model by attenuating neuroinflammation and gliosis when the genetic insult was combined with the external inflammatory insult. The EP2 antagonist treatment was not effective in either single-hit—genetic, or in single-hit—environmental models.

## Materials and methods

### Animals

**Fig. 1:**
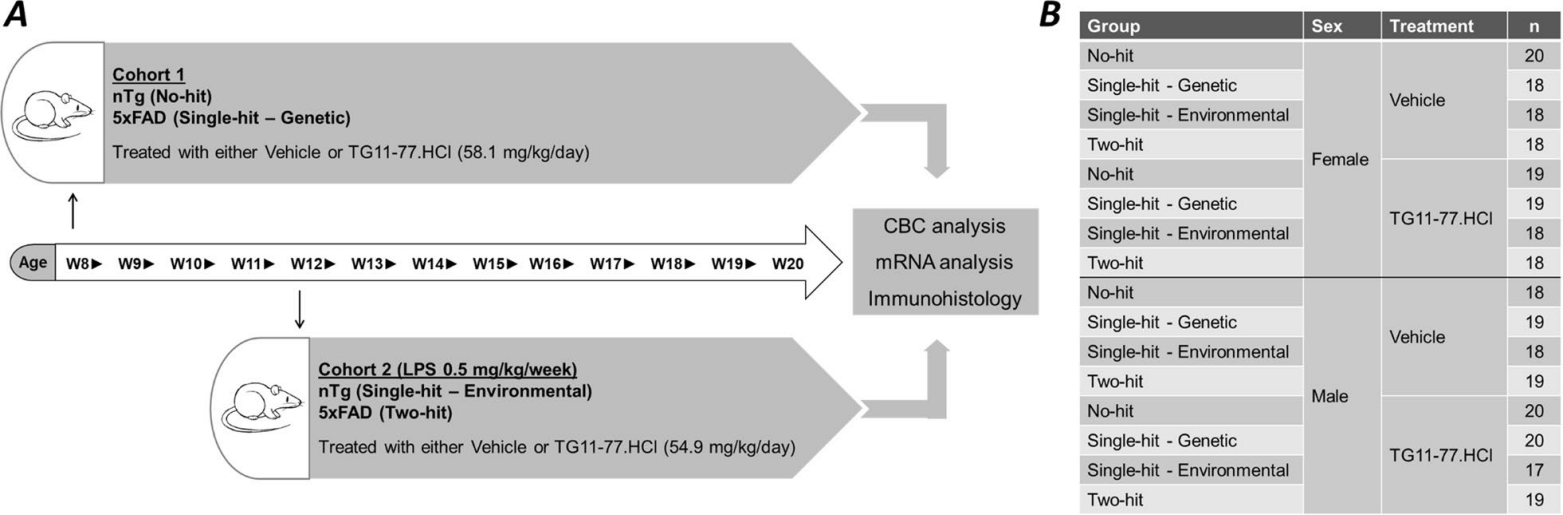
Experimental paradigm of LPS and TG11-77.HCl administration in 5xFAD mice. **A** Both males and females along with their non-transgenic (nTg) littermates were separated into two cohorts in the study. Animals were perfused with PBS 24 h after the last LPS injection. For further analysis, blood and brain were harvested before and after perfusion, respectively. **B** Table showing all the groups and their sex, treatment and number (*n*) used in the study

We used a 5xFAD transgenic mouse model of AD in this study. These mice are developed by overexpressing two mutant human proteins, i.e., amyloid β precursor protein 695 (APP) and presenilin 1 (PS1), both implicated to be involved in AD pathology. 5 familial AD (FAD) mutations (APP: K670N, M671L (Swedish), I716V (Florida), V717I (London) and PS1: M146L and L286V) are inserted in the transgene construct and regulated through the neuron specific promoter Thy1 for selective overexpression in the brain. This model exhibits AD related pathologies including amyloid plaques, glial activation in different regions of the brain as early as 2–3 months of their age and neuronal loss at 9 months of age [50]. The 5xFAD line was originally created on the C57BL6/SJL genetic background (MMRRC Stock No: 34840-JAX). However, this line was subsequently backcrossed over ten generations on a congenic C57BL/6 background (MMRRC Stock No: 34848-JAX) and used by others. We received a pair of congenic 5xFAD males on C57BL/6 from our colleague Dr. Malu Tansey at Emory University. The 5xFAD males were mated with non-transgenic (labeled as “nTg” hereafter) females to generate colonies and added into the experiments at the appropriate age. Both 5xFAD and nTg mice were housed together in standard auto water cages under 12 h light/dark cycle with ad libitum access to food and water. Mice were transferred to manual water bottles when they were introduced to the drug treatment. Two cohorts of mice were used in the experiments (Fig. 1A). Cohort 1 comprised the no-hit (nTg) and single-hit (genetic) model from 8 to 20 weeks of age. Cohort 2 included a single-hit (environmental) and a two-hit (genetic and environmental) model that were injected once per week with lipopolysaccharide **(**LPS; L2880, Sigma, USA) from 12 to 20 weeks of age. In total 153 mice were included in cohort 1 and 145 mice in cohort 2 (both sexes, 5xFAD and nTg) (Fig. 1B). Body weight, weekly water consumption and modified Irwin scores were measured for all mice once every week.

### Modified Irwin test

A modified Irwin test was performed on all the mice from both single-hit and two-hit cohorts weekly once along the length of the treatment. Mice were scored to access their health based on seven physical or behavioral parameters as per the method previously described [51]. Ptosis, lacrimation, body posture, tremor, dragging body, hyperactivity or hypoactivity and coat appearance were measured on a scale of 0–2, where 0 = normal; 1 = mild-to-moderate impairment; 2 = severe impairment. The range of scores was 0–14.

### Drug administration

Lipopolysaccharide (LPS) (Sigma; L2880) was dissolved in sterile saline and injected intraperitoneally (IP) weekly once at 0.5 mg/kg of body weight. TG11-77.HCl, an EP2 antagonist recently developed and synthesized in our laboratory [48], was prepared fresh every week by dissolving in rodent drinking water at 0.5 mg/mL. For optimum solubility of the drug, the solution was adjusted to pH 3.5 using 1 M HCl. Drinking water without TG11-77.HCl adjusted to pH 3.5 was used as the vehicle. Drug or vehicle was given to mice *ad-libitum* in graduated glass drinking water bottles (Ancare, USA) and the volume measured weekly once to calculate drug consumption.

### Tissue processing

Mice were anesthetized with isoflurane and terminally perfused with ice-cold saline 24 h after the last LPS injection in cohort 2. Before perfusion, blood samples were collected. Brains were harvested after perfusion, longitudinally bisected and appropriately stored for further processing. The neocortex and hippocampus were dissected from one half brain, quickly transferred to dry ice and then stored at − 80 °C until gene expression analysis. The other half of the brain was fixed in 4% paraformaldehyde in PBS (Sigma) and stored in 4 °C until processed for immunohistochemical analysis.

### Complete blood count (CBC) analysis

Fresh blood was collected by cardiac puncture using 1 ml syringes with 21G needles, transferred to EDTA tubes and stored at 4 °C until sent for CBC analysis. CBC analysis was performed within 24 h of collection by the Quality Assurance and Diagnostic Lab, Division of Animal Resources, Emory University using a VetScan HM5 v2.3 Hematology Analyzer. Sixteen different blood cell parameters were analyzed, including differential white blood cells (WBC), red blood cells (RBC) and platelet counts, distribution width for different cell types, levels of hemoglobin and hematocrit.

### qRT-PCR

As previously described [52], 10–13 frozen neocortex tissues from each group were homogenized using a sonicator in 1 ml RNA extraction buffer supplied with the Quick-RNA MiniPrep Kit (Zymo Research). Homogenized tissue samples were centrifuged at 500 g for 1 min to settle the tissue debris. An aliquot of 700 µl of the homogenate was used for RNA extraction as per the manufacturer’s protocol. RNA samples were quantified using an Epoch Microplate Spectrophotometer (BioTek) and further converted to cDNA using qScript cDNA SuperMix (Quanta Biosciences). Quantitative real-time polymerase chain reaction (qRT-PCR) was performed in a CFX96 Touch Real-Time PCR Detection System (Bio-Rad) using iQ SYBR Green Supermix (Quanta Biosciences) with each sample run in technical duplicates. Three housekeeping genes, β-actin, glyceraldehyde-3-phosphate (GAPDH), and hypoxanthine phosphoribosyl transferase 1 (HPRT1) were used as internal controls. mRNA expression for 5 pro-inflammatory mediators, [interleukin-6 (IL-6), C–C motif chemokine ligands 2 (CCL2), tumor necrosis factor alpha (TNF), interleukin 1-beta (IL1β) and prostaglandin E2 receptor (EP2)] and 4 glial markers [ionized calcium binding adaptor molecule (Iba1), glial fibrillary acidic protein (GFAP), marker for infiltrating macrophages (CD11b) and S100 calcium-binding protein B (S100B)] were tested. For qRT-PCR analysis, cycle threshold (CT) values for each gene of interest were normalized to their respective geometric means of CT values from all 3 housekeeping genes. The fold changes in the treatment groups were measured using the 2^−ΔΔCt^ method by calculating relative expression from the respective control groups [53]. The fold changes were used for statistical analysis between groups. The primer sequences for different genes used for qRT-PCR are listed in Table 1.

**Table 1:** Mouse primer sequences used in qRT-PCR reactions

Genes	Forward Primer (sequence 5′–3′)	Reverse Primer (sequence 5′–3′)
βActin	AAGGCCAACCGTGAAAAGAT	GTGGTACGACCAGAGGCATAC
GAPDH	TGTCCGTCGTGGATCTGAC	CCTGCTTCACCACCTTCTTG
HPRT1	GGAGCGGTAGCACCTCCT	CTGGTTCATCATCGCTAATCAC
IL-6	TCTAATTCATATCTTCAACCAAGAGG	TGGTCCTTAGCCACTCCTTC
CCL2	CATCCACGTGTTGGCTCA	GCTGCTGGTGATCCTCTTGTA
TNF	TCTTCTGTCTACTGAACTTCGG	AAGATGATCTGAGTGTGAGGG
IL1β	TGAGCACCTTCTTTTCCTTCA	TTGTCTAATGGGAACGTCACAC
EP2	TCTTTAGTCTGGCCACGATGCTCA	GCAGGGAACAGAAGAGCAAGGAGG
Iba1	GGATTTGCAGGGAGGAAAAG	TGGGATCATCGAGGAATTG
GFAP	GACAACTTTGCACAGGACCTC	ATACGCAGCCAGGTTGTTCT
CD11b	CCAGTAAGGTCATACAGCATCAGT	TTGATCTGAACAGGGATCCAG
S100B	TCGGACACTGAAGCCAGAG	AGACATCAATGAGGGCAACC

### Immunohistochemistry

The brain hemisphere fixed in 4% paraformaldehyde (Sigma) was transferred to 30% sucrose (Sigma) for at least 48 h and then moved to phosphate buffered saline (PBS) (Sigma) before sending to the Neuropathology/ Histochemistry Core of the Emory NINDS Neurosciences Core Facility for tissue section. As previously described [51], 7–10 brain samples from each group were processed as per the facility protocol and each alternate 10 µm paraffin embedded coronal section was collected along the entire length of hippocampus using a microtome (Leica). For immunofluorescent staining, rabbit polyclonal to GFAP (Abcam, ab16997, 1:500 dilution) and Iba1 (Wako, 019-19741, 1:500 dilution) primary antibodies were used. For secondary antibody, Alexa Fluor goat anti-rabbit 488 (Thermofisher, A11008, 1:1000 dilution) was used. Briefly, sections mounted on glass slides were deparaffined in xylenes for 10 min and then hydrated in gradually decreasing concentrations of ethanol (100, 95, 75, 50% in water). For antigen retrieval, slides were boiled in antigen retrieval solution (DAKO) at 98 ºC for 20 min and then slowly cooled to RT for 15 min. To block nonspecific binding, samples were incubated in a solution containing 5% goat serum and 0.3% Triton-X in PBS for at least 1 h and then incubated with primary antibody (diluted in 2% goat serum and 0.3% Triton-X in PBS) overnight at 4 °C. Then sections were washed with PBS (3 × for 5 min each) and subsequently incubated for 2 h at room temperature in secondary antibody (diluted in 1% goat serum and 0.3% Triton-X in PBS). The sections were again washed with PBS. Finally, the sections were stained for Congo red (see below), or mounted with DAPI mounting media (Vectra Shield) for nuclear staining. The fluorescent images were captured with an AxioObserver A1 fluorescence microscope (Zeiss) using the AxioVision AC 4.7 software (Zeiss). Same illumination intensity and image acquisition parameters were used to capture all the images across different treatments. We restricted the staining to female treatment groups only as qRT-PCR analysis showed the effect of drug only in female brains. 7–12 mice from each group and for each mouse 4 equidistance sections (every 20th section) from one hemisphere were selected for staining. Specific anatomical markers were used to ensure that the images were obtained from the same regions of the sections to reduce variability. We intended to focus on different regions of brain hemi cortex, thalamus and hippocampus for immunohistochemical quantification. Hence, we captured the brain sections under 20× magnified objectives. We have also statistically analyzed the data obtained from different regions to show any effect of the TG11-77.HCl treatment on immunoreactivity and overall size and number of the amyloid plaques.

### Congo red staining

Four equidistant sections (every 20th section) from one hemisphere were selected for staining and measured for an average plaque count per mouse (comprising average numbers from 4 sections in each mouse) with 12–16 brain samples from each group. As described previously [52], after secondary antibody staining, brain sections were immediately transferred to 0.2% Congo red (Sigma) in saturated ethanolic NaCl solution [52]. 1 mL of activator (1% NaOH) was freshly added to 100 mL of pre-staining solution A (2.5% NaCl solution in 80% Ethanol) or solution B (0.2% Congo Red in 80% ethanolic NaCl solution) before use. Sections were incubated in solution A for 25 min and then incubated in solution B for 40 min at room temperature. Finally, the slides were washed with PBS for 10 min and quickly destained with 80% and 50% ethanol for 1 min each to remove excess stain from the section. After a final wash with PBS the slides were mounted with DAPI mounting media (Vectra Shield) for nuclear staining. Congo red positive plaques appear bright red using a rhodamine filter on the fluorescent microscope. The images were captured in AxioObserver A1 fluorescence microscope (Zeiss) using the AxioVision AC 4.7 software (Zeiss). Same illumination intensity and image acquisition parameters were used to capture all the images across different treatments.

### Image quantification

Green fluorescent images from Iba1 or GFAP positive sections and red fluorescent images from Congo red stained sections were analyzed using ImageJ software. Total number of particles, size and total area covered by fluorescence in each section were quantified. Each TIFF image was converted to an 8-bit binary (black and while) image and subjected to auto thresholding using one of the 16 built in threshold filters in ImageJ. The same filter was used throughout. Using the “Analyze particles” feature of the software, the number, size and area were measured and further analyzed for graphical representation.

### Statistical analysis

Data were statistically analyzed in GraphPad Prism 9. For body weight and Irwin test, two-way repeated measure ANOVA with Sidak’s multiple comparisons test was applied. For CBC analysis, multiple unpaired student’s *t* test with Bonferroni correction was applied between groups. For LPS effect on mRNA we used one-way ANOVA with Dunnett’s multiple comparisons test. For mRNA and immunohistochemical analysis, paired *t* test was applied between groups to estimate an overall effect of the treatment. For individual genes and markers analysis we used two-way ANOVA with Tukey’s multiple comparisons test and for individual brain regions in immunohistochemical quantification we used unpaired student’s *t* test with multiple comparisons using two-stage step-up (Benjamini, Krieger, and Yekutieli) with FDR (Q) = 5%. Data are represented as Mean ± SEM for each group. The percent change in the expression of all markers in qRT-PCR is calculated by the formula: ((Mean2/Mean1) − 1) × 100 in single-hit—environmental model and ((Mean2 − 1/Mean1 − 1) − 1) × 100 in two-hit model, where Mean1 represents the vehicle treated group and Mean2 represents TG11-77.HCl treated group. The percent change in immunohistological quantification is calculated by the formula: (Mean difference/Mean 1) × 100.

## Results

As shown in Fig. 1B, sixteen groups of mice were included. Mice in cohort 1 had both sexes of 5xFAD (single-hit—genetic) and their non-transgenic (nTg) littermates (no-hit) treated with either EP2 antagonist TG11-77.HCl (58.1 mg/kg/day) or its vehicle (drinking water at pH 3.5) for 12 weeks starting at 8 weeks of age. Cohort 2 comprised both sexes of 5xFAD (two-hit) and nTg (single-hit—environmental) mice administered LPS (0.5 mg/kg/week ip) for 8 weeks as a second hit to induce additional brain inflammation, and treated with TG11-77.HCl (54.9 mg/kg/day) or the vehicle, starting at 12 weeks until 20 weeks of age (Fig. 1A). The duration and start times of the drug in both the cohorts vary. Cohort 1 mice were treated for 3 months beginning 8 weeks till 20 weeks of their age. The neuroinflammatory signaling was weak in these cohorts (see details below). The outcome from this cohort along with a previous study reporting once a week LPS injection for 2 months [54] guided us to delay the start time in cohort 2 until 12 weeks, and only treat them for 2 months. The data from males and females were analyzed separately, because we previously identified a sex difference in neuroinflammation in 5xFAD mice [52]. We dosed the EP2 antagonist to these mice at a nominal rate of 100 mg/kg/day. Therefore, based on a projected average consumption of daily water (~ 5 mL) by a 25 g mouse, we prepared the drug solution at 0.5 mg/mL concentration in drinking water. However, to determine the actual dose of free base of the drug delivered to these mice we measured weekly the body weight (Additional file 1: Fig. S1) and volume consumed (Additional file 11: Table S1). Based on measured 92.5% recovery of the drug from drinking water after 7 days at room temperature, the rate of free base of the drug consumption was calculated as shown in Additional file 11: Table S1. There was no effect of the transgene, or treatment with LPS or EP2 antagonist TG11-77.HCl, on weight gain of mice from 8 to 20 week period (Additional file 1: Fig. S1), or on measures of behavioral and physical parameters by a modified Irwin test [51], (Additional file 2: Fig. S2).

### LPS induces anemia of inflammation in blood and neuroinflammation in neocortex of both two-hit 5xFAD mice and single-hit—environmental nTg mice

**Fig. 2:**
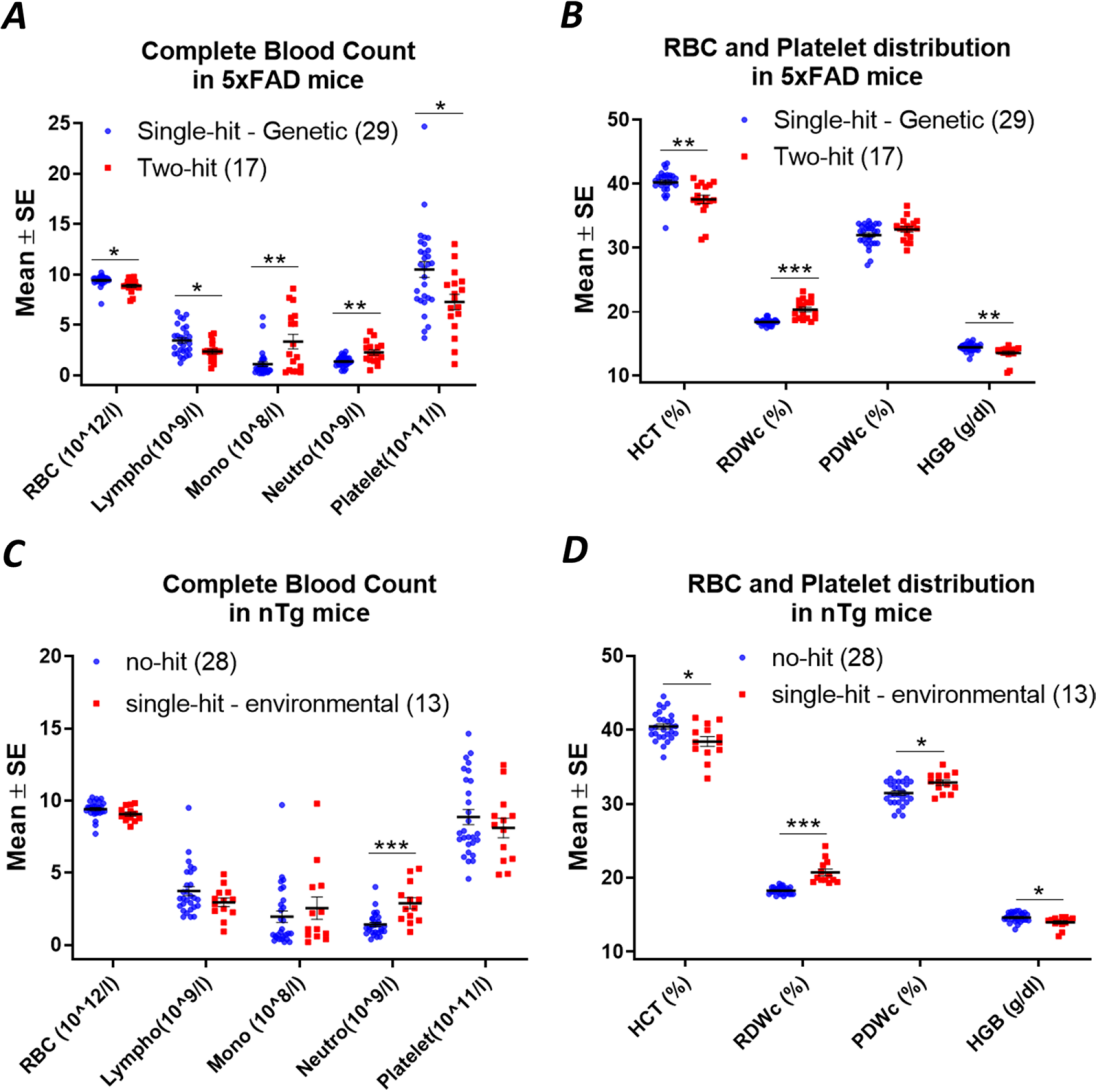
LPS induces anemia of inflammation in blood cells of both 5xFAD (two-hit) and nTg (single-hit—environmental) mice. Mice of both sexes were administered with LPS (0.5 mg/kg, intraperitoneal, weekly, ip) from 12 to 20 weeks of age. Mice were euthanized 24 h after the last LPS injection. **A** Effect of LPS on RBC, lymphocyte, monocyte, neutrophil and platelet counts in two-hit 5xFAD mice, compared to single-hit—genetic mice. **B** Effect of LPS on hemoglobin (HGB), % hematocrit (HCT) and distribution width of RBC (RDWc) and platelet (PDWc) in two-hit 5xFAD mice. **C** Effect of LPS on blood cell count in single-hit—environmental mice, compared to no-hit mice. **B** Effect of LPS on hemoglobin (HGB), % hematocrit (HCT) and cell distribution width in single-hit—environmental mice. Multiple unpaired *t* test with Bonferroni correction was applied between groups. *P* values were set to be significant at * ≤ 0.05, ** ≤ 0.01 and *** ≤ 0.001. Data are mean ± SEM

**Fig. 3:**
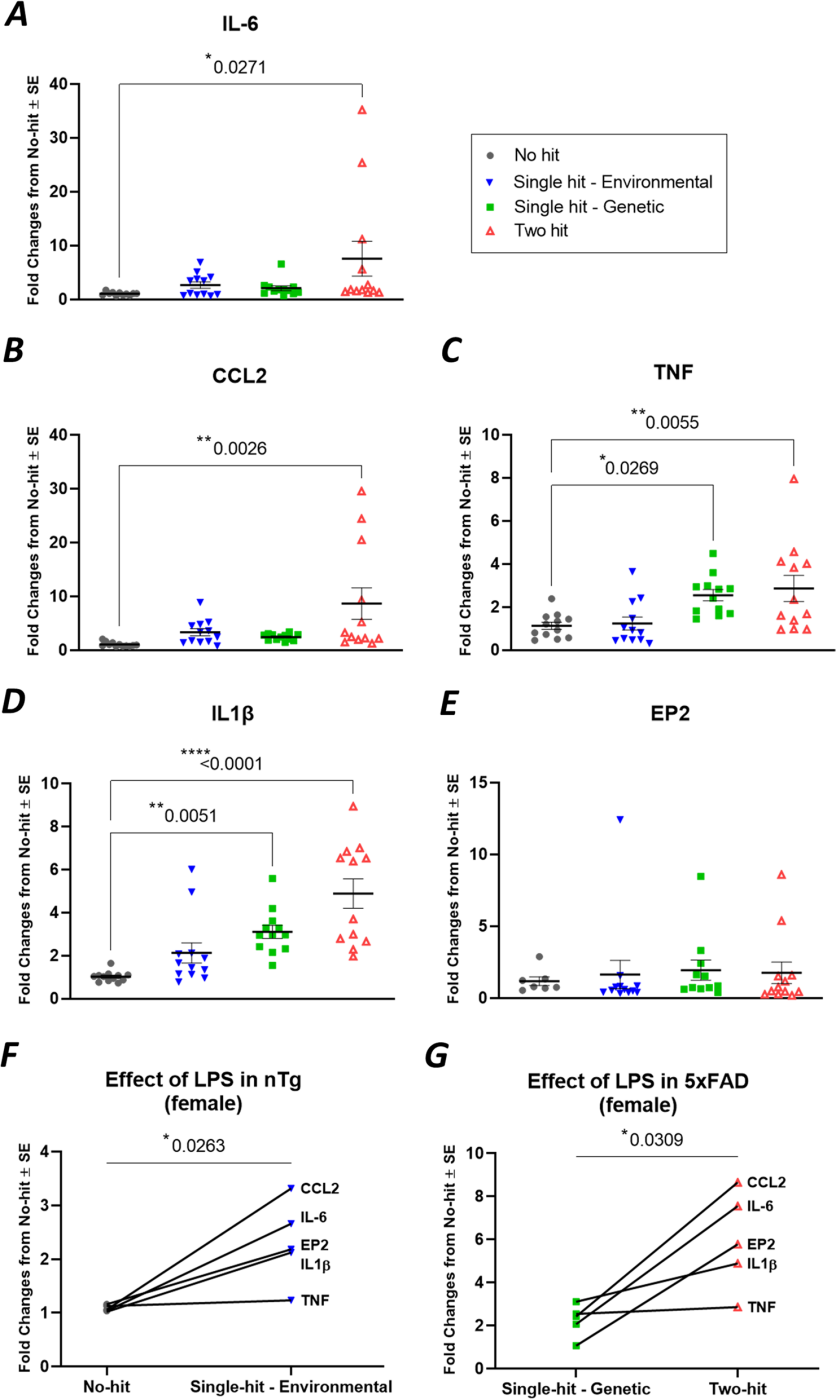
LPS induces selective neuroinflammation in neocortex of two-hit 5xFAD females. **A**–**E** The mRNA fold changes of individual proinflammatory mediators in all hits (environmental and/or genetic) compared to no-hit mice. **F** LPS induced elevation in the group of proinflammatory mediators in single-hit—environmental mice from no-hit mice. **G** LPS induced elevation in the group of proinflammatory mediators in two-hit mice from single-hit—genetic mice. All the groups were normalized to no-hit mice. For individual endpoint analysis one-way ANOVA with Dunnett’s multiple comparisons test was applied (**A**–**E**). For group analysis between different hits, paired *t-*test was applied for the series of pro-inflammatory genes (**F**, **G**). *P* values were set to be significant at * ≤ 0.05, ** ≤ 0.01 and **** ≤ 0.0001. Data are mean ± SEM

**Fig. 4:**
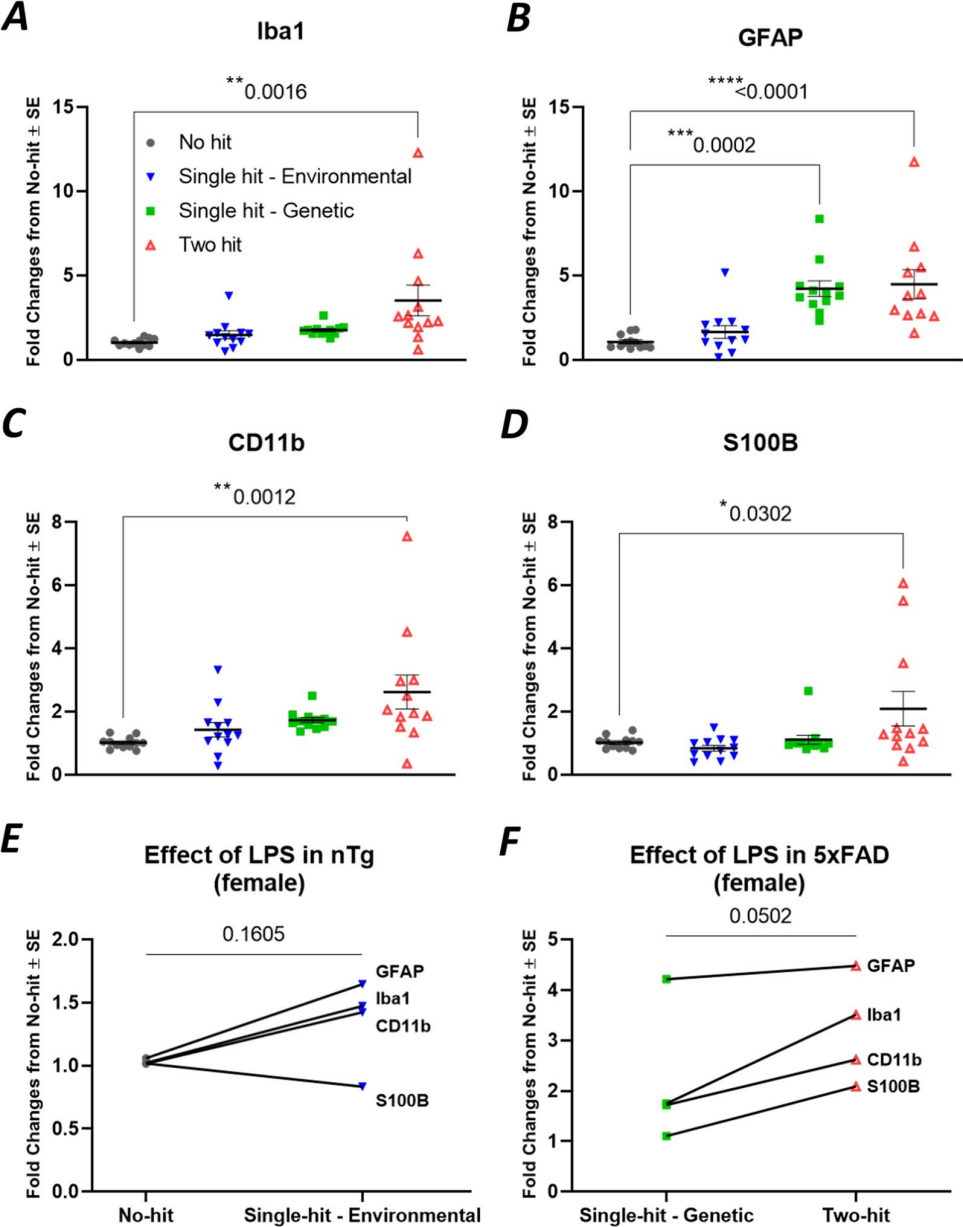
LPS induces gliosis in neocortex of two-hit 5xFAD females. **A**–**D** mRNA fold changes of individual astroglial and microglial markers in all hits (environmental and/or genetic) compared to no-hit mice. **E** LPS induced elevation in the group of glial markers in single-hit—environmental mice from no-hit mice. **F** LPS induced elevation in the group of glial markers in two-hit mice from single-hit—genetic mice. All the groups were normalized to no-hit mice. For individual endpoint analysis one-way ANOVA with Dunnett’s multiple comparisons test was applied (**A**–**D**). For group analysis between different hits, paired *t* test was applied for the series of pro-inflammatory genes (**E**, **F**). *P* values were set to be significant at * ≤ 0.05, ** ≤ 0.01 and **** ≤ 0.0001. Data are mean ± SEM

LPS was intraperitoneally injected once a week (0.5 mg/kg) into 5xFAD mice (termed two-hit) and their nTg littermates (termed single-hit—environmental) from 12 to 20 weeks of age. A total of 9 injections of LPS were given to these mice. Complete blood cell (CBC) analysis 24 h after the last LPS dose revealed that the numbers of red blood cells (RBCs), lymphocytes and platelets were significantly reduced, while monocytes and neutrophils were significantly increased in two-hit 5xFAD mice (Fig. 2A). Furthermore, the levels of hemoglobin (HGB) and % hematocrit (HCT) were significantly reduced, whereas RBC width (RDWc) (significant) and platelet distribution width (PDWc) (nonsignificant) were increased (Fig. 2B). These changes in the number and size of RBCs, white blood cells (WBCs) and platelets are typical of “anemia of inflammation” [55] and demonstrate that LPS treatment produced a mild systemic response. LPS injection induced a similar response in single-hit—environmental nTg mice but not as much as observed in two-hit 5xFAD mice (Fig. 2C, D). mRNA encoding pro-inflammatory (IL-1β, TNF, IL-6, CCL2) mediators, astroglial and microglial markers (IBA1, GFAP, CD11b, S110B), were significantly elevated individually and also as a group in neocortex by LPS treatment in two-hit 5xFAD female mice (Figs. 3, 4). Similarly, pro-inflammatory mediators and astroglial and microglial markers were also elevated in LPS treated nTg female (single-hit—environmental) neocortex (Figs. 3, 4). In males, LPS induced similar inflammatory response as seen in females. LPS induced elevation in pro-inflammatory mediators and astroglial–microglial markers was found in single-hit—environmental and two-hit male neocortex (Additional file 3: Fig. S3, Additional file 4: Fig. S4).

### EP2 antagonist (TG11-77.HCl) treatment had no effect on blood counts or LPS-induced anemia of inflammation

We determined whether the EP2 antagonist has an effect on the CBC parameters in either nTg (no-hit) or 5xFAD (single-hit—genetic) mice without LPS treatment. Combined together (nTg and 5xFAD), there were no alterations in blood cell counts or distribution upon TG11-77.HCl treatment in the single hit cohort, suggesting the drug has no adverse effect on the peripheral system of 5xFAD mice and their nTg littermates (Additional file 5: Fig. S5A, B). Moreover, the number of RBCs, WBCs and platelets and levels of hemoglobin (HGB), % hematocrit (HCT), red blood cell (RDWc) and platelet distribution width (PDWc), which were altered following chronic LPS administration, were unchanged upon TG11-77.HCl treatment in the cohort of 5xFAD (two-hit) and nTg (single-hit—environmental) mice together (Additional file 5: Fig. S5C, D). We have also examined 5xFAD and nTg mice separately in both cohorts and confirmed similar unresponsiveness to TG11-77.HCl treatment as combined together (data not shown). This suggests that the drug has no effect on the LPS induced “anemia of inflammation” in either two-hit or single-hit—environmental cohort.

### EP2 antagonist TG11-77.HCl attenuates mRNA levels encoding proinflammatory and glial proteins in the cortex of two-hit female mouse AD model

**Fig. 5:**
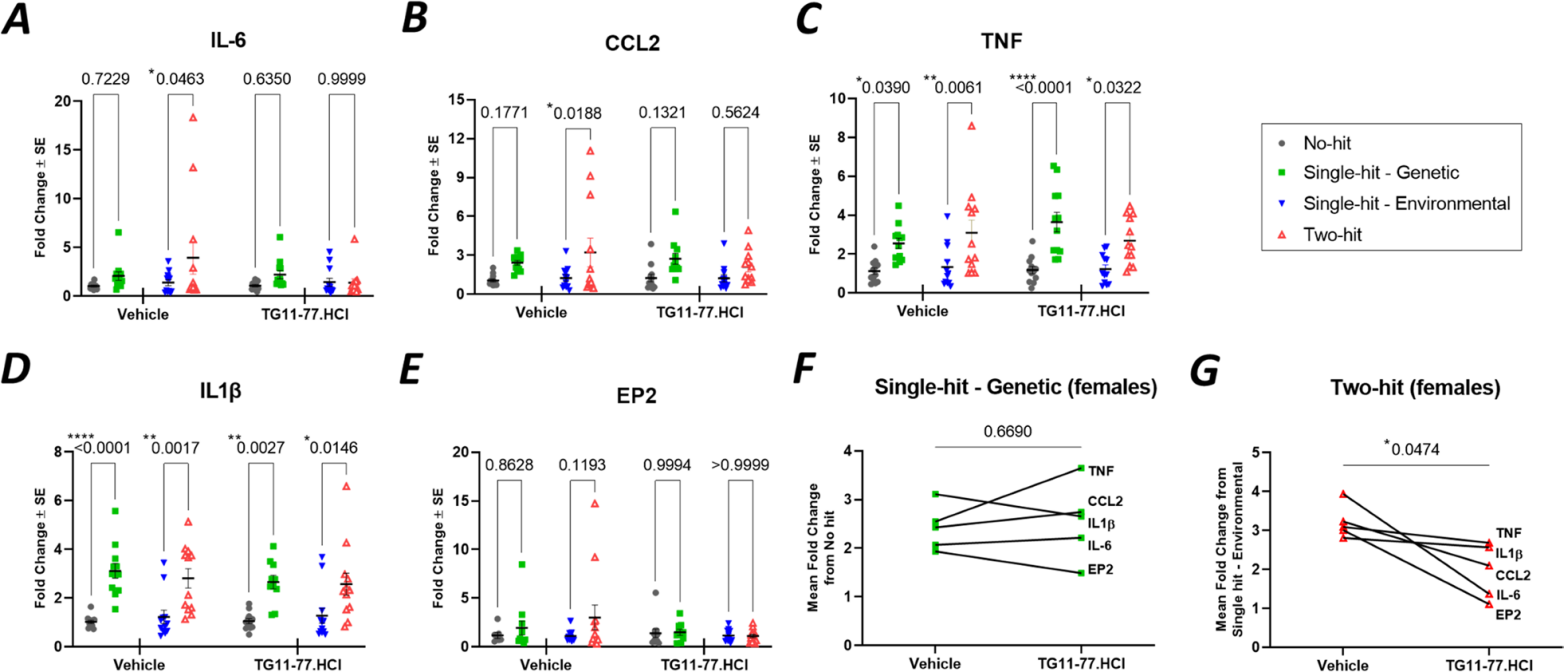
Selective anti-inflammatory effect of EP2 antagonist in two-hit 5xFAD females. **A**–**E** Effect of single-hit (genetic) and two-hit (genetic and environmental) on neuroinflammatory markers in brain neocortex of 5xFAD female mice treated with or without EP2 antagonist TG11-77.HCl. The fold changes in 5xFAD groups (single-hit—genetic or two-hit) were normalized to their respective nTg groups (no-hit or single-hit—environmental). **F** Pairwise effect of TG11-77.HCl treatment in single-hit—genetic females. **G** Pairwise effect of TG11-77.HCl treatment in two-hit females. For individual endpoint analysis two-way ANOVA (hit, treatment) with Tukey’s multiple comparisons test was applied (**A**–**E**). For group analysis between vehicle and TG11-77.HCl treatment among different hits, paired *t* test was applied for the series of pro-inflammatory genes (**F**, **G**). *P* values were set to be significant at * ≤ 0.05, ** ≤ 0.01 and **** ≤ 0.0001. Data are mean ± SEM

**Fig. 6:**
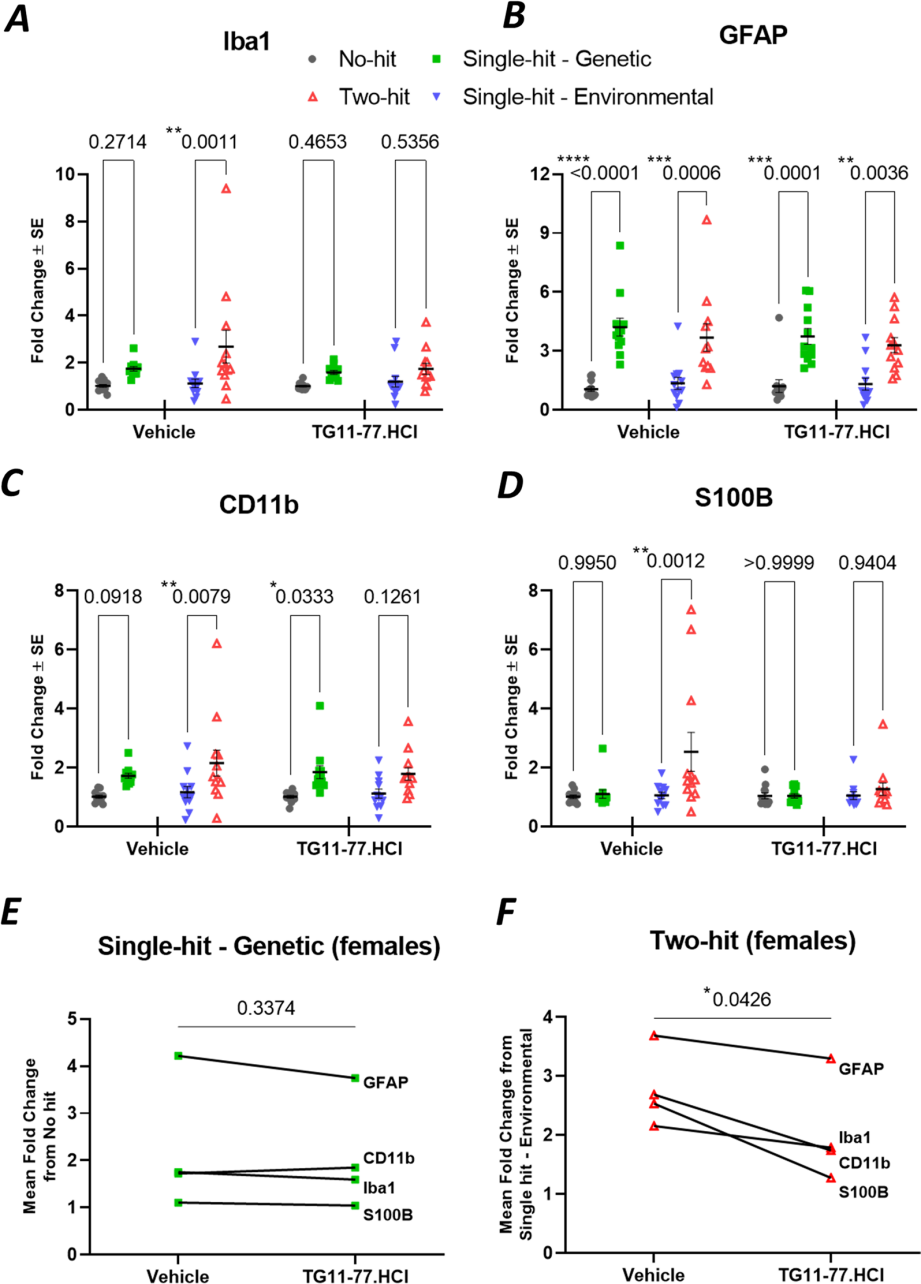
Selective glial inhibition by EP2 antagonist treatment in two-hit 5xFAD females. **A**–**D** Effect of single-hit (genetic) and two-hit (genetic and environmental) on astroglial and microglial markers in brain neocortex of 5xFAD female mice treated with or without EP2 antagonist TG11-77.HCl. The fold changes in 5xFAD groups (single-hit—genetic or two-hit) were normalized to their respective nTg groups (no-hit or single-hit—environmental). **E** Pairwise effect of TG11-77.HCl treatment in single-hit—genetic females. **F** Pairwise effect of TG11-77.HCl treatment in two-hit females. For individual endpoint analysis two-way ANOVA (hit, treatment) with Tukey’s multiple comparisons test was applied (**A**–**D**). For group analysis between vehicle and TG11-77.HCl treatment among different hits, paired *t* test was applied for the series of glial genes (**E**, **F**). *P* values were set to be significant at * ≤ 0.05, ** ≤ 0.01, *** ≤ 0.001 and **** ≤ 0.0001. Data are mean ± SEM

The levels of proinflammatory mediators (IL-1β, TNF, IL-6, CCL2, EP2) and astroglial–microglial markers (IBA1, GFAP, CD11b, S100B) were upregulated in 5xFAD mice and these effects were higher in females compared to males, confirming previous findings [52]. LPS induced a more robust neuroinflammation in female two-hit 5xFAD cortex (Figs. 3, 4) compared to males (Additional file 3: Fig. S3, Additional file 4: Fig. S4). To determine whether EP2 antagonism can suppress chronic neuroinflammation in a two-hit model of 5xFAD brains, we first analyzed cortical tissue from TG11-77.HCl treated mice for mRNA expression. Chronic treatment with EP2 antagonist TG11-77.HCl for 8 weeks significantly attenuated (paired *t* test, *P* < 0.05) levels of these inflammatory (Fig. 5G) and astroglial–microglial markers (Fig. 6F) in LPS treated (two-hit) 5xFAD females but not in LPS treated (two-hit) 5xFAD males (Additional file 6: Fig. S6G, Additional file 7: Fig. S7F). By contrast, TG11-77.HCl treatment did not ameliorate the inflammatory effect in the single-hit—genetic cohort of 5xFAD female (Figs. 5F, 6E) or male mice (Additional file 6: Fig. S6F, Additional file 7: Fig. S7E).

To analyze drug effect in each 5xFAD group, the mRNA fold changes were normalized to their respective nTg groups (single-hit—genetic normalized to no-hit and two-hit normalized to single-hit—environmental) to show relative expression from their respective controls. From these, the proinflammatory mRNA levels were found to be reduced by an average of 53% by TG11-77.HCl, and the astroglial–microglial mRNAs by 46% (see Methods for equations used). When looked at individually, vehicle treatment did not reduce the LPS induced upregulation of all pro-inflammatory mediators in two-hit females, while TG11-77.HCl lowered all of them, i.e., IL-6 (87%), CCL2 (51%), TNF (20%), IL-1β (13%) and EP2 (95%) (Fig. 5A–E). Similarly in astroglial–microglial markers, Iba1 (56%), GFAP (15%), CD11b (32%) and S100B (82%) were alleviated upon TG11-77.HCl treatment in two-hit females (Fig. 6A–D). This alleviation was not found in most of the pro-inflammatory and glial markers in single-hit—genetic females upon TG11-77.HCl treatment (Figs. 5, 6). This result indicates that the EP2 antagonist exerted a selective anti-inflammatory effect only in the two-hit model when there was elevated neuroinflammation in 5xFAD mice induced by the secondary environmental hit, LPS.

To determine whether TG11-77.HCl exerts an anti-inflammatory effect after LPS treatment in nTg brain, we have also tested the drug in single-hit—environmental mice. The mRNA folds of proinflammatory mediators and astroglial–microglial markers in single-hit—environmental neocortex was found unchanged in both males and females after TG11-77.HCl treatment compared to vehicle. Rather, TG11-77.HCl treatment caused an increase (*P* < 0.05, paired *t* test) in glial markers (overall 56%) in male mice of single-hit—environmental cohort (Additional file 8: Fig. S8).

### TG11-77.HCl attenuates LPS induced astrogliosis and microgliosis in different brain regions of two-hit 5xFAD females

**Fig. 7:**
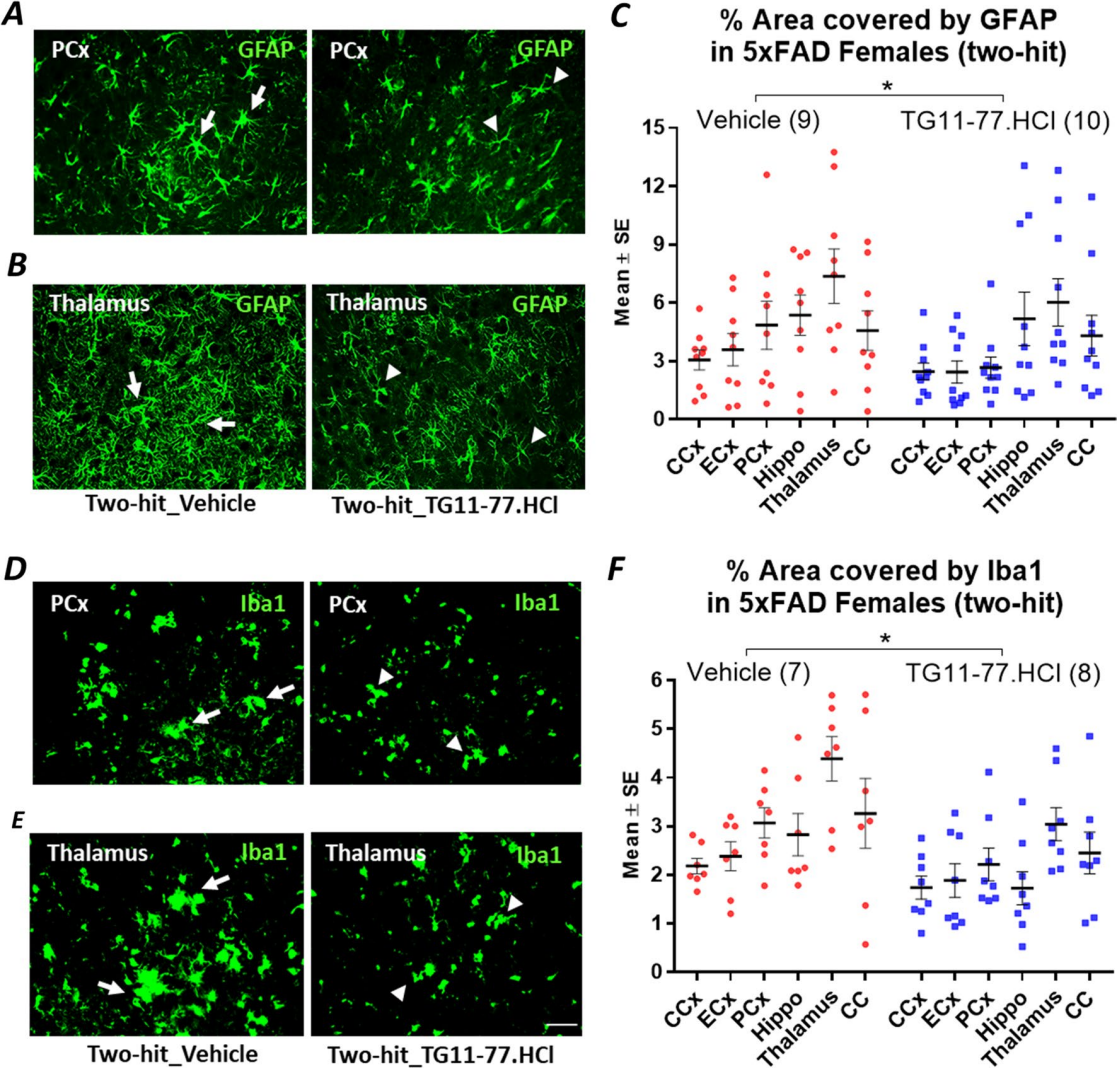
Selective inhibition of GFAP and Iba1 immunoreactivity by EP2 antagonist in two-hit 5xFAD females. Effect of EP2 antagonist TG11-77.HCl treatment in different regions of the brain on expression of astroglial protein, GFAP (**A**–**C**) and microglial protein, Iba1 (**D**–**F**). Representative microscopic images are presented showing area covered by the proteins in TG11-77.HCl treated mouse brain regions (arrowheads) compared to Vehicle treated mice (arrows) in piriform cortex (**A**, **D**) and thalamus (**B**, **E**). % area at different brain regions were calculated from the microscopic images using a standard protocol in ImageJ (**C**, **F**). Paired *t* test was applied between groups and multiple unpaired *t* test with FDR (5%) was applied for multiple comparison. *P* values were set to be significant at * ≤ 0.05. Scale bar = 50 mm. Data are mean ± SEM. *CCx* cerebral cortex, *ECx* entorhinal cortex, *PCx* piriform cortex, *Hippo* hippocampus, *CC* corpus callosum

To determine the effect of TG11-77.HCl on gliosis, we examined astrocytic (GFAP) and microglial (Iba1) proteins by immunohistochemistry in brain sections of (two-hit) LPS induced 5xFAD females. Paraffin embedded brain sections from one hemisphere from LPS injected female mice were stained for GFAP and Iba1 immunoreactivity. Images were captured under a 20X objective and analyzed in ImageJ to measure % area covered by GFAP and Iba1 positive cells in different regions of the brain. Representative microscopic images revealed a significant reduction in the area covered by GFAP (overall 21% reduced, *P* < 0.05) (Fig. 7C) and Iba1 (overall 27% reduced, *P* < 0.05) (Fig. 7F) in TG11-77.HCl treated two-hit 5xFAD female brain regions (arrowheads showing smaller cell bodies) compared to vehicle treated 5xFAD females (arrows showing larger cell bodies) (Fig. 7A, B, D, E). This beneficial effect of EP2 antagonist against glial activation was observed in most brain regions, including cerebral cortex, entorhinal cortex, piriform cortex, thalamus, hippocampus and corpus callosum in the two-hit cohort. However, we did not find this effect of EP2 antagonist in the single-hit—genetic female 5xFAD cohort, as there were fewer GFAP and Iba1 positive cells found in the brain of single-hit cohort (overall 2% reduction in GFAP and 11% in Iba1 stained area, Additional file 9: Fig. S9) when compared to the brain of two-hit cohort (Fig. 7). Together these data confirm the anti-inflammatory effect of the EP2 antagonist TG11-77.HCl, by suppression of gliosis and neuroinflammation only in the two-hit model of 5xFAD, where genetic and environmental insults reinforce each other.

### EP2 antagonist, TG11-77.HCl increases amyloid plaque deposition in different brain regions of two-hit female 5xFAD mice

**Fig. 8:**
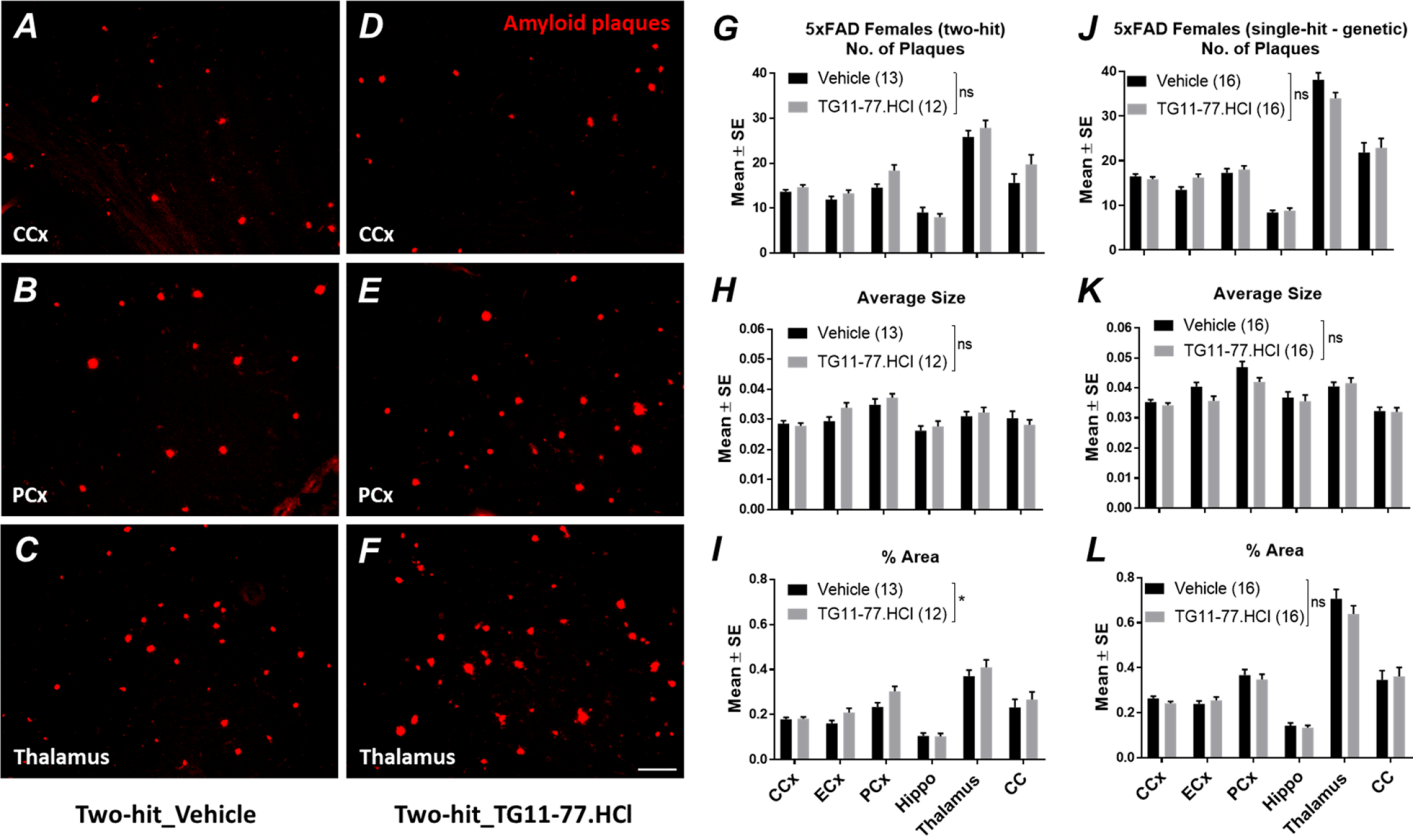
EP2 antagonist treatment increases amyloid deposition in two-hit 5xFAD female brain. Different brain regions of the 5xFAD females from two-hit and single-hit—environmental cohorts treated either with vehicle or TG11-77.HCl are stained with Congo red. Microscopic images reveal numbers and size of amyloid plaques in vehicle (**A**–**C**) and TG11-77.HCl treated mice (**D**–**F**). ImageJ quantification demonstrates number, size and % area covered by the plaques in different regions of the brain after TG11-77.HCl treatment in two-hit (**G**–**I**) and single-hit genetic mice (**J**–**L**). Paired *t* test was applied between groups and multiple unpaired *t* test with FDR (5%) was applied for multiple comparison. *P* values were set to be significant at * ≤ 0.05. Scale bar = 50 mm. Data are mean ± SEM. *CCx* cerebral cortex, *ECx* entorhinal cortex, *PCx* piriform cortex, *Hippo* hippocampus, *CC* corpus callosum

To further examine the effect of TG11-77.HCl treatment on amyloid pathology, we have measured the total number of Congo red (CR) positive amyloid plaques, their average size and area covered by plaques in different regions of the brain from two-hit 5xFAD female mice treated either with vehicle or TG11-77.HCl (Fig. 8). Representative images from different brain regions (Fig. 8A–F) revealed that there is a trend towards increasing number and size of the Congo red plaques in cerebral cortex (CCx), piriform cortex (PCx) and thalamus (Fig. 8G, H), and a modest, but statistically significant (*P* < 0.05, paired *t* test) increase in % area (13%) covered by the Congophilic plaques in the two-hit 5xFAD female mice treated with TG11-77.HCl compared to vehicle (Fig. 8I). However, when analyzed for multiple comparisons, there was no significant difference found in any of the brain regions independently. In addition, there was no statistically significant effect of TG11-77.HCl on the overall number (3% increase), size (4% decrease) and % area (3% decrease) covered by the plaques in single-hit—genetic 5xFAD female brains without the LPS insult (Fig. 8J–L), confirming the effect of drug was restricted to two-hit cohort of the 5xFAD brains.

### TG11-77.HCl shows high selectivity to EP2, and oral dosing of TG11-77.HCl achieves adequate concentrations in plasma and brain

**Fig. 9:**
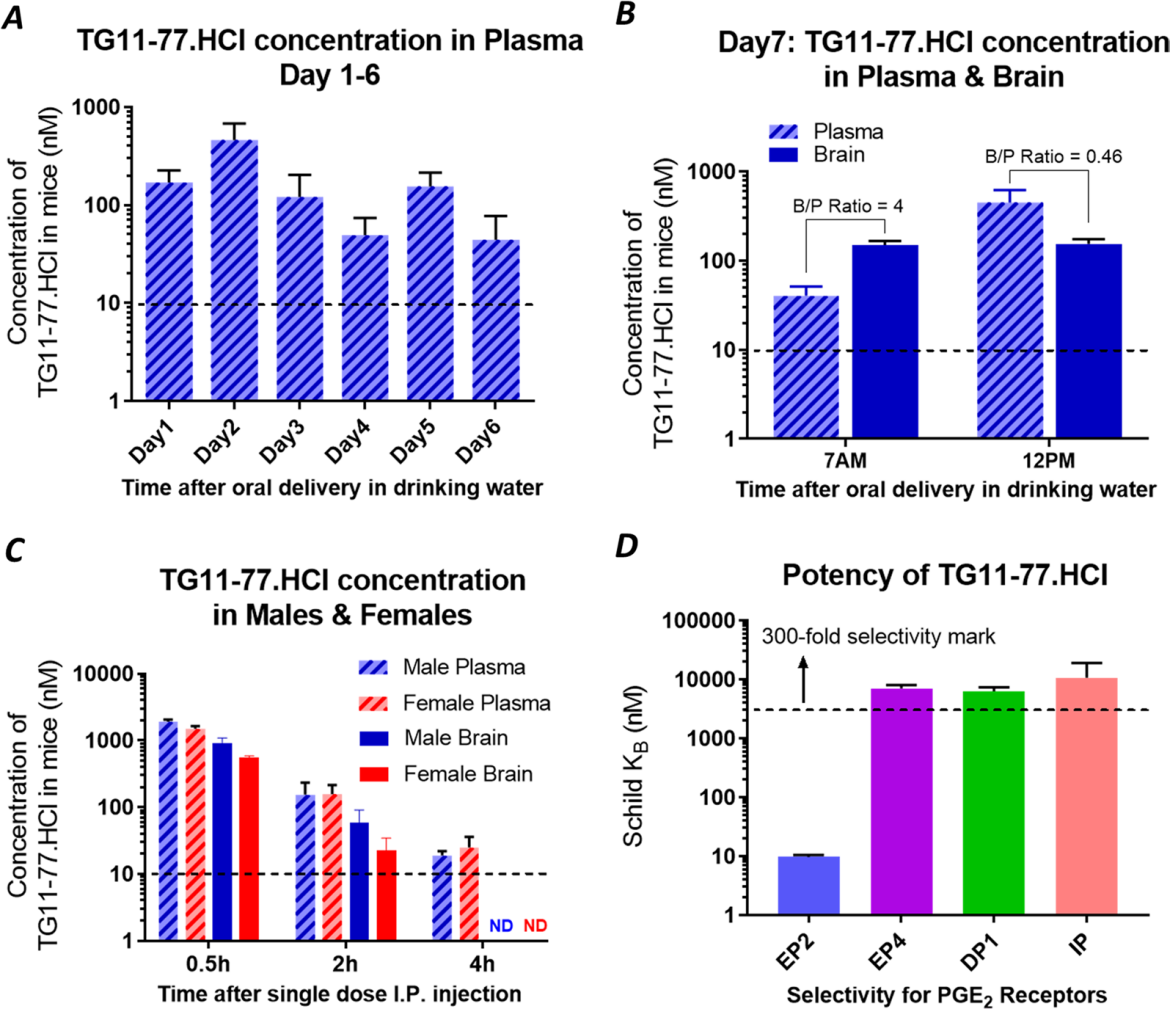
TG11-77.HCl is highly selective to EP2 receptor, and achieves adequate plasma and brain concentrations upon oral dosing. **A** Plasma concentrations of TG11-77.HCl in 8–12-week-old female C57BL/6 mice (*n* = 4) by oral dosing from day 1 to 6 (with projected dose of 100 mg/kg/day). Schild K_B_ is indicated by dotted line. **B** On day 7, the concentration in the brain was measured with a brain-to-plasma ratio 0.46–4 depending on the timing of the analysis. Dotted line in both **A** and **B** represents the potency of TG11-77.HCl (Schild KB = 9.7 nM). **C** Plasma concentration with increasing timepoints (0.5, 2 and 4 h) after single dose IP injection of TG11-77.HCl (10 mg/kg) in male and female C57BL/6 mice (*n* = 3). ND: concentrations not detected. Dotted line represents the potency (Schild K_B_) of TG11-77.HCl = 9.7 nM. **D** EP2 Selectivity was determined by measuring the potency of TG11-77.HCl for other prostanoid receptors (EP4, DP1 and IP) by a cAMP mediated TR-FRET assay using human EP2 expressed C6-gloma cells as shown before [49]

To determine whether chronic oral dosing of TG11-77.HCl in drinking water achieves a requisite concentration in plasma and the brain tissue, we delivered TG11-77.HCl to 8–12 weeks old C57BL6 mice, via drinking water at the strength of 0.5 mg/mL. This concentration produces a target dose of 100 mg/kg/day based on the body weight and average daily consumption of water by a mouse. Blood samples were collected from a pool of 8 mice, from which plasma samples were processed for further analysis. On days 1–6 after dosing began, we found an average 17-fold higher concentration of TG11-77 free base in plasma, than its EP2 potency (Schild K_B_ = 9.7 nM) of the free base (Fig. 9A) [49]. In the same cohort of mice (*n* = 4), on day 7 the concentration of TG11-77 in the plasma varied between 4.2 and 46.5-fold higher than its potency and it was 15–16-fold higher in the brain than its potency. TG11-77.HCl showed a brain-to-plasma ratio between 0.46 and 4 depending on the timing of the blood draw from these mice (Fig. 9B; the brain level remained constant, while the plasma levels changed. Assuming free drug equals measured drug concentration in the brain given very low protein content of CSF, these data suggest that chronic oral dosing in drinking water delivers sufficient concentration of TG11-77.HCl to the brain to occupy approximately 93% of EP2 receptors in the absence of agonist. Furthermore, we have tested a single dose intraperitoneal injection of TG11-77.HCl (at 10 mg/kg) in separate groups of male and female C57BL/6 mice (*n* = 3), to understand whether the distribution of the compound over time in the plasma and brain varies between the sexes. Two hours after intraperitoneal injection, about 16-fold higher plasma concentration compared to the Schild potency (K_B_) of the compound was recorded in both males and females (Fig. 9C). Although TG11-77.HCl achieved a similar plasma concentration in both the sexes, its concentration in male brain was found to be sixfold higher compared to only twofold higher in female brain, in comparison to the potency of the molecule (Fig. 9C). Nonetheless, these data suggest males and females had adequate exposure to TG11-77HCl either by chronic oral dosing, or by intraperitoneal injection. To determine whether TG11-77.HCl could inhibit other prostanoid receptors, we tested the potency of the TG11-77.HCl against EP4, DP1 and IP receptors, which are all Gs-coupled GPCRs and structurally 30–44% similar to EP2 [34, 56]. TG11-77.HCl showed significantly lower potency against DP1, EP4 and IP receptors compared to EP2 receptor (> 300-fold selectivity) (Fig. 9D). Moreover, when tested against other prostanoid receptors in binding assays, TG11-77.HCl showed non-significant inhibition (< 50%) of target-ligand (radiolabeled PGE_2_ for EP1 and EP3 or BW245C for DP1) binding even at high (10 µM) concentrations, whereas it inhibited 92% of radiolabeled PGE_2_ to EP2 at 1 µM (Additional file 10: Fig. S10), indicating TG11-77.HCl as a selective EP2 targeting agent.

## Discussion

Due to numerous failures in recent clinical trials designed to test many anti-amyloid drugs, there has been a renewed focus on neuroinflammation in AD as a potential target for developing disease modifying therapeutics [57]. However, availability of rodent models with neuroinflammation comparable to clinical cases of AD in human is limited [58]. A two-hit hypothesis has been proposed indicating multiple pathological markers and interlinking signaling cascades may initiate AD pathology in the brain [59, 60]. However, two-hit models that exhibit Aβ-plaques and neuroinflammation by external insult have not yet been explored to examine whether an anti-inflammatory treatment will have any therapeutic benefit in altering disease pathology. To test this hypothesis, we aimed to examine the anti-inflammatory efficacy of a novel EP2 antagonist in a two-hit model of AD, where external environmental stimuli induced by LPS exacerbates the innate inflammatory cascade in 5xFAD mice. For comparison, we also tested a single-hit—genetic cohort of 5xFAD mice without LPS injection, and another single-hit—environmental cohort of nTg mice in which LPS was administered to create a chronic low-grade inflammation.

The findings from our study reveal a chronic dose of LPS induces chronic anemia of inflammation in blood and elevates brain inflammation in both two-hit 5xFAD (Fig. 2A, B) and single-hit—environmental nTg cohorts (Fig. 2C, D). However, an anti-inflammatory effect of the EP2 antagonist was recorded only in two-hit female model of AD, in which genetic and environmental insults were combined together, suggesting that EP2 drives neuropathology in a two-hit model of AD in which an external environmental stimulus exacerbates brain inflammation compared to a simple genetic model (single-hit) (Figs. 5, 6). The antagonist treatment beginning at prodromal stage of AD was not found to be effective in either of the single-hit models of genetic (Figs. 5, 6) or environmental (Additional file 8: Fig. S8) predisposition. Interestingly, the LPS mediated chronic anemia of inflammation in blood is not altered by chronic treatment with EP2 antagonist TG11-77.HCl, either in the non-transgenic (single-hit—environmental) or 5xFAD (single-hit—genetic and two-hit) mice (Additional file 5: Fig. S5), indicating reduction of neuroinflammation by TG11-77.HCl in two-hit 5xFAD mice most likely reflects a CNS action through suppression of EP2 mediated neuroinflammation. A recent study demonstrates that inhibition of myeloid EP2 signaling rejuvenates cellular bioenergetics, systemic and brain inflammatory states, synaptic plasticity and spatial memory, and, blockade of peripheral myeloid EP2 signaling is sufficient to restore cognition in aged mice [61]. It would be interesting to investigate whether suppression of neuroinflammation by our selective EP2 antagonist will offer any benefits on neurodegeneration and cognitive impairment in AD models.

Earlier, LPS has been used to induce a mild-to severe inflammatory reaction in the periphery as well in the brain [23, 62, 63]. The impact of LPS in rodents largely depends on the dose, route and interval of the injection. High doses of LPS are associated with severe injury and mortality in rodents, but a low dose has been shown to induce a mild chronic anemia of inflammation in the blood and brain [64–66]. The proof-of-concept tests presented in our study involve administration of a chronic moderate dose of LPS (0.5 mg/kg/week for 8 weeks) in a transgenic model of 5xFAD mice (two-hit) and their nTg littermates (single-hit—environmental) (Fig. 1). In both two-hit and single-hit—environmental model, LPS has an anemia-like effect in the blood (Fig. 2), with reduction in RBCs, lymphocytes and platelet numbers, hemoglobin and % hematocrit, and increase in number of monocytes and neutrophils. Interestingly, LPS exacerbates the brain inflammation in two-hit 5xFAD female mice (165% mean increase in pro-inflammatory markers and 44% mean increase in glial markers) compared to single-hit—environmental mice (113% mean increase in pro-inflammatory markers and 31% mean increase in glial markers) (Figs. 3G, 4F). This exacerbation was similar in male two-hit mice upon LPS insult (pro-inflammatory markers: 158% and 160% increase in single-hit—environmental and two-hit, respectively; glial markers: 92% and 94% increase in single-hit—environmental and two-hit, respectively) (Additional file 3: Fig. S3G, Additional file 4: Fig. S4F). Although percent increase in pro-inflammatory and glial markers in both the sexes remain similar upon LPS injection, the effect of transgene was always found to be higher in females worsening the overall induction in two-hit females. These data suggest that a chronic environmental insult can worsen the neuroinflammation in AD brain facilitating a two-hit model of the disease to test anti-inflammatory drugs. The incidence of Alzheimer’s Disease is higher in women than men, and this is not due to longer longevity of women [67]. The larger inflammatory response of female mice [52] (and this study) is likely to contribute to the gender difference in AD incidence.

Furthermore, chronic treatment with a potent and selective EP2 antagonist, TG11-77.HCl (54.9 mg/kg/day for 8 weeks) significantly attenuated the mean mRNA expression of proinflammatory mediators in neocortex by 53% and astroglial–microglial markers by 46% only in female 5xFAD mice from two-hit cohort (Figs. 5G, 6F). Multiple comparisons reveal 87% reduction in IL-6 (Fig. 5A) and 51% reduction in CCL2 (Fig. 5B) markers in two-hit 5xFAD females upon TG11-77.HCl treatment. This observation further supports our previous finding from an in-vitro assay, where the same EP2 antagonist significantly suppressed an EP2 agonist induced mRNA elevation of IL-6 cytokine along with IL‐1β [49]. In the current study, the reduction in CCL2 also strongly suggests an anti-inflammatory role of TG11-77.HCl, which can be attributed to the critical role of CCL2 in monocyte infiltration and associated brain inflammation in a mouse model of status epilepticus demonstrated through activation of EP2 signaling in our previous studies [68]. It would be interesting to see if infiltration of monocytes adds to inflammation in the two-hit 5xFAD brain and whether our EP2 antagonist could protect the brain from this insult. We have recently reported that a chemically distinct EP2 antagonist when systemically delivered, abolishes monocyte entry after SE [68]. Similarly 46% overall reduction of astroglial–microglial mRNA markers (56% in Iba1 and 82% in S100B) upon TG11-77.HCl treatment in two-hit 5xFAD (Fig. 6A, D) confirms the anti-inflammatory properties of this drug possibly through suppression of microglial activation in the inflamed brain, which was further verified by reduction in percent area covered by glial markers, such as GFAP (21%) and Iba1 (27%) in different areas of the brain from two-hit 5xFAD females (Fig. 7). This effect was not found in single-hit—genetic 5xFAD females (Additional file 9: Fig. S9) suggesting the drug was only effective to address underlying neuroinflammation in AD brain when it was subjected to an additional environmental inflammatory insult by LPS (two-hit).

Even though our previous work showed a sex difference in neuroinflammation in 5xFAD mice [52], it is still unclear how LPS induced inflammation varies between sex and transgene. Therefore, additional studies are warranted to determine why TG11-77.HCl treatment was not effective in the male mice of two-hit cohort of 5xFAD. One possible reason could be the two-hit 5xFAD males (Additional file 3: Fig. S3, Additional file 4: Fig. S4) did not show as large an inflammatory induction as two-hit 5xFAD females (Figs. 3, 4). Another possibility is that the drug may have been metabolized differently in both sexes. To address this latter possibility, we examined the concentration of TG11-77.HCl in brain and plasma by single intraperitoneal injection of 10 mg/kg. We found similar drug concentrations in both male and female mice plasma over 0.5–4 h (Fig. 9C). Moreover, there was about 16-fold higher concentration in both female and male plasma at 2 h timepoint; 2.3-fold higher in female brain, and sixfold higher in male than the Schild potency of the molecule (Fig. 9C) indicating a possibly slower metabolism of the drug in the males. Overall, the pharmacokinetics data suggest the efficacy of the EP2 antagonist should be found in both the sexes. Nonetheless, the anti-inflammatory effects of TG11-77.HCl in the two-hit 5xFAD females is congruent with our recent findings in acute brain injury models of status epilepticus (SE), in which a short-term treatment of EP2 antagonist significantly attenuated the upregulation of inflammatory genes and glial markers in SE mice and rats [69–72].

It is interesting to note that our EP2 antagonist, TG11-77.HCl did not exert an anti-inflammatory effect in chronic LPS injected single-hit—environmental nTg mice. However, we recently showed that an acute treatment of another EP2 antagonist, TG6–10–1 mitigates neuroinflammation in a single high-dose (3 mg/kg) LPS-injected sepsis-associated encephalopathy (SAE) model [62]. Although, we cannot compare SAE model with the chronic low-dose injected anemic single-hit nTg model, it is worth mentioning that in SAE model the induction of cytokines and chemokines was very high (100-fold for IL-1β, CCL2, TNF; > 75 fold for IL-6) 6 h after LPS-injection, indicating a full-blown inflammatory condition in the brain cortex by a single acute dose of LPS, whereas in our single-hit—environmental nTg mice multiple chronic dosing of LPS (0.5 mg/kg/week) induced modest but long term inflammatory induction. In future studies, it would be worthwhile to investigate a chronic LPS dose higher than 0.5 mg/kg/week to more robustly increase brain inflammation by engaging EP2 mediated inflammatory pathways to achieve a higher therapeutic effect of our candidate drug.

Although the direct involvement of EP2 signaling in neurodegenerative processes is still under investigation, recent reports indicate that global deletion of EP2 in APPSwe (APP-PS1) mice [45] or conditional deletion of EP2 in microglia reduces Aβ-burden, functional and spatial memory deficits, suggesting EP2 impairs the beneficial function of microglia [46]. It has also been demonstrated that microglia from EP2 ablated mice showed enhanced phagocytosis and reduced neurotoxicity by Aβ [44]. Taken together, these studies reinforce the hypothesis that the EP2 receptor, in particular expressed on microglia or monocytes [68], modulates glial activation and negatively impacts the homeostatic functions of microglia in removing debris, such as Aβ from the brain, and exacerbates the priming of microglia leading to an inefficient inflammatory state. However, in our study, the chronic treatment of EP2 antagonist did not culminate in enhanced clearance of Aβ plaques from brain cortex of two-hit 5xFAD females (Fig. 8A–I). In contrary, chronic TG11-77.HCl treatment increased the % area covered by Aβ-plaques in LPS induced two-hit 5xFAD brains (Fig. 8I), suggesting that reduced activation of glial cells upon chronic TG11-77.HCl treatment may lead to reduced microglial and astroglial mediated phagocytosis and subsequent clearance of Aβ (Fig. 8J–L). Although there was no effect found on the amyloid pathology in single-hit—genetic 5xFAD females upon TG11-77.HCl treatment. Nonetheless, the downstream mechanisms by which the EP2 receptor modulates the phagocytic activity of microglia is not clear, because either stimulation of EP2 receptors with an agonist or antagonist had no impact on the phagocytic activity of microglia BV2 cell line that overexpressed human EP2 receptors [48], in comparison to enhanced phagocytosis found in primary mouse microglia lacking EP2 receptors [44].

Recently, we have developed several potent and selective EP2 antagonists with an aim to investigate their anti-inflammatory properties in CNS disorders [49, 69]. From this, TG11-77.HCl emerged as a lead candidate with high potency (Schild K_B_ = 9.7 nM) and > 300-fold selectivity towards EP2 receptor against other structurally close prostanoid receptors DP1, IP, EP4, EP1, and EP3 (Fig. 9D and Additional file 10: Fig. S10), suggesting that chronic oral dosing of TG11-77.HCl will not achieve sufficient plasma or brain concentrations to inhibit these other receptors. Therefore, effects we found with TG11-77.HCl can likely be attributed to EP2 receptor antagonism. This antagonist showed a brain-to-plasma ratio ranging from 0.4 to 4 (Fig. 9A, B), and no adverse toxicity by chronic dosing ≥ 100 mg/kg (Rawat et al. in preparation). It also displayed anti-inflammatory properties in vitro in a BV2 cell line overexpressing human EP2 receptor [48]. Moreover, this compound is soluble in water at concentration of 1.1 mg/mL, which facilitates chronic oral dosing in drinking water. The two-hit mouse model might better represent the majority of Alzheimer’s patients who consume a “western diet”, which has been shown to promote chronic inflammation driven by innate immunity [73]. Previous studies have shown how a first hit (exposure to environmental Lead (Pb) through diet at infancy) triggered epigenetic dysregulation that led to AD-like pathogenesis in aged *Macaca* monkeys [22, 74], suggesting the value of two-hit models to understand the pathology of a complex disease, such as AD.

## Conclusion and prospects

**Fig. 10:**
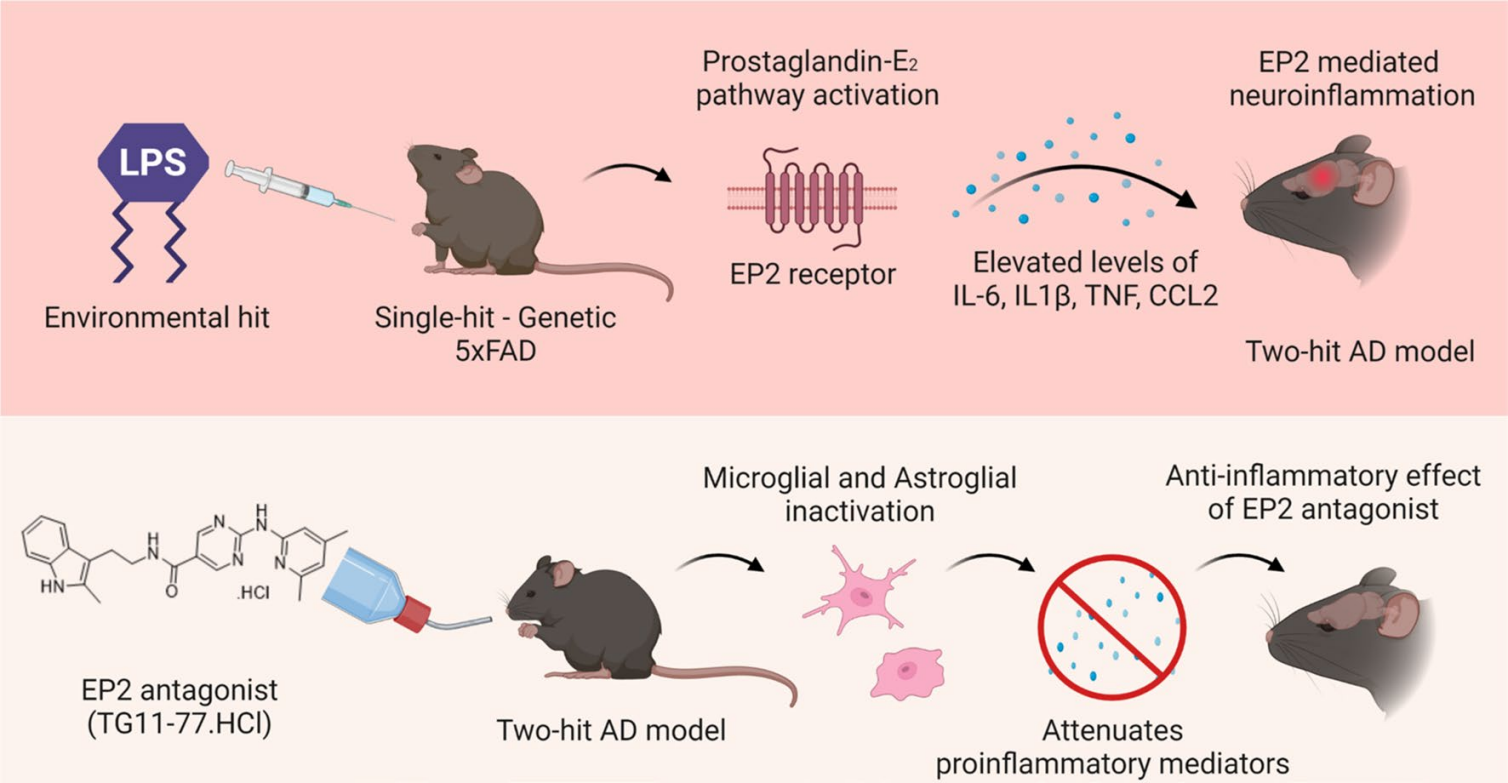
Schematic showing plausible mechanism behind the study outcome. Top panel shows how LPS may induce EP2 mediated neuroinflammation in two-hit AD brains, where both single-hits (environmental and genetic) act in synergy. Bottom panel shows how EP2 receptor antagonist (TG11-77.HCl) ameliorates neuroinflammation selectively in the two-hit AD brains

A major conclusion is that a selective EP2 antagonist treatment is found to be anti-inflammatory in a two-hit model of 5xFAD, where transgene and a second hit of environmental insult might have triggered EP2 derived neuroinflammation in the AD brain (Fig. 10). Interestingly, EP2 antagonist did not attenuate LPS induced anemia of inflammation in these mice. Although the antagonist did not exert any beneficial effects on the amyloid burden in AD brains, it will be worth looking at tau pathology and associated inflammation in future experiments. We project that future studies are warranted to elucidate the pathways involved in triggering neuroinflammation and associated pathophysiology in the two-hit model of AD. Furthermore, optimization of treatment regimen at varying age of the disease progression should also be investigated in this model.

## Supplementary Information

**Additional file 1: Fig. S1.** No adverse effect of transgene, LPS and TG11-77.HCl on body weight gain in different cohorts of mice. The % body weight gain was measured weekly once from week 8 to week 20 in cohort 1 (no-hit and single-hit—genetic) (A) and from week 12 to week 20 in cohort 2 (single-hit—environmental and two-hit) mice (males and females combined) (B). (C) % weight gain upon TG11-77.HCl treatment in no-hit (C) and single-hit—genetic mice (D). Two-way repeated measure ANOVA with Sidak’s multiple comparisons test was applied. No statistical significance between groups was found on these A–D measures. Data are mean ± SEM.

**Additional file 2: Fig. S2.** No adverse effect of TG11-77.HCl treatment on behavioral and physical activities. Modified Irwin scores showing a cumulative score (range 0–14) from seven behavioral parameters in the mice from both two-hit (A, B) and single-hit cohorts (C, D) treated either with vehicle or TG11-77.HCl. Two-way repeated measure ANOVA with Sidak's multiple comparisons test was applied. No statistical significance between groups was found on these A–D measures. Data are mean ± SEM.

**Additional file 3: Fig. S3.** LPS induces neuroinflammation in neocortex of two-hit 5xFAD males. (A–E) mRNA fold changes of individual proinflammatory mediators in all hits (environmental and/or genetic) compared to no-hit mice. (F) LPS induced elevation in the group of proinflammatory mediators in single-hit—environmental mice from no-hit mice. (G) LPS induced elevation in the group of proinflammatory mediators in two-hit mice from single-hit—genetic mice. All the groups were normalized to no-hit mice. For individual endpoint analysis one-way ANOVA with Dunnett’s multiple comparisons test was applied (A–E). For group analysis between different hits, paired t test was applied for the series of pro-inflammatory genes (F, G). P values were set to be significant at * =  < 0.05, ** =  < 0.01, *** =  < 0.001 and **** =  < 0.0001. Data are mean ± SEM.

**Additional file 4: Fig. S4.** LPS induces gliosis in neocortex of two-hit 5xFAD males. (A–D) mRNA fold changes of individual astroglial and microglial markers in all hits (environmental and/or genetic) compared to no-hit mice. (E) LPS induced elevation in the group of glial markers in single-hit—environmental mice from no-hit mice. (F) LPS induced elevation in the group of glial markers in two-hit mice from single-hit—genetic mice. All the groups were normalized to no-hit mice. For individual endpoint analysis one-way ANOVA with Dunnett’s multiple comparisons test was applied (A–E). For group analysis between different hits, paired t test was applied for the series of pro-inflammatory genes (F, G). P values were set to be significant at * ≤ 0.05, ** ≤ 0.01 and *** ≤ 0.001. Data are mean ± SEM.

**Additional file 5: Fig. S5.** EP2 antagonist does not reverse LPS induced anemia of inflammation. (A, B). Complete blood count (CBC) and cell distribution analysis in no-hit and single-hit—genetic combined cohort, upon TG11-77.HCl treatment. (C, D) CBC counts and distribution in single-hit—environmental and two-hit mice combined in presence or absence of TG11-77.HCL treatment. Multiple unpaired t test with Bonferroni correction was applied between groups. P values were set to be significant at * =  < 0.05, but no significance between groups was found on these A–D measures. Data are mean ± SEM.

**Additional file 6: Fig. S6.** No anti-inflammatory effect of EP2 antagonist in single-hit and two-hit 5xFAD males. (A–E) Effect of single-hit (genetic) and two-hit (genetic and environmental) on neuroinflammatory markers in brain neocortex of 5xFAD male mice treated with or without EP2 antagonist TG11-77.HCl. The fold changes in 5xFAD groups (single-hit—genetic or two-hit) were normalized to their respective nTg groups (no-hit or single-hit—environmental). (F) Pairwise effect of TG11-77.HCl treatment in single-hit—genetic males. (G) Pairwise effect of TG11-77.HCl treatment in two-hit males. For individual endpoint analysis two-way ANOVA (hit, treatment) with Tukey's multiple comparisons test was applied (A–E). For group analysis between vehicle and TG11-77.HCl treatment among different hits, paired t-test was applied for the series of pro-inflammatory genes (F, G). P values were set to be significant at * ≤ 0.05 and **** ≤ 0.0001. Data are mean ± SEM.

**Additional file 7: Fig. S7.** No effect of EP2 antagonist on glial markers in single-hit and two-hit 5xFAD males. (A–D) Effect of single-hit (genetic) and two-hit (genetic and environmental) on astroglial and microglial markers in brain neocortex of 5xFAD male mice treated with or without EP2 antagonist TG11-77.HCl. The fold changes in 5xFAD groups (single-hit—genetic or two-hit) were normalized to their respective nTg groups (no-hit or single-hit—environmental). (E) Pairwise effect of TG11-77.HCl treatment in single-hit—genetic males. (F) Pairwise effect of TG11-77.HCl treatment in two-hit males. For individual endpoint analysis two-way ANOVA (hit, treatment) with Tukey’s multiple comparisons test was applied (A–D). For group analysis between vehicle and TG11-77.HCl treatment among different hits, paired t test was applied for the series of glial genes (E, F). P values were set to be significant at * ≤ 0.05 and ** ≤ 0.01. Data are mean ± SEM.

**Additional file 8: Fig. S8.** No effect of EP2 antagonist on proinflammatory mediators and glial markers in single-hit—environmental nTg mice. Effect of TG11-77.HCL treatment on mRNA level of proinflammatory mediators in single-hit—environmental nTg female (A) and male brain neocortex (C). Effect of TG11-77.HCL treatment on astroglial and microglial markers in single-hit—environmental nTg females (B) and males (D). The fold changes in single-hit—environmental groups were normalized to their respective nTg groups (no-hit), represented by dotted line in the graphs. For group analysis between vehicle and TG11-77.HCl treatment, paired t test was applied. P values were set to be significant at * ≤ 0.05. Data are mean ± SEM.

**Additional file 9: Fig. S9.** TG11-77.HCl did not alter GFAP and Iba1 immunoreactivity in single-hit—genetic 5xFAD female brains. Representative images from TG11-77.HCl or vehicle treated brains showing GFAP immunoreactivity in (A) PCx and (B) thalamus and (C) % area covered in different regions of the brain analyzed by imageJ. Iba1 immunoreactivity in (D) PCx and (E) thalamus and (F) % area covered in different brain regions from single-hit genetic females. Paired t test was applied between groups and multiple unpaired t test with FDR (5%) was applied for multiple comparison. P values were set to be significant at * ≤ 0.05. Scale bar = 50 mm. Data are mean ± SEM. CCx: cerebral cortex; ECx: entorhinal cortex; PCx: piriform cortex; Hippo: hippocampus; CC: corpus callosum.

**Additional file 10: Fig. S10.** TG11-77.HCl exhibits highly selective inhibition of EP2 receptor. Percent inhibition of different radiolabeled prostanoid agonists (PGE2 to EP1–3, or BW245C to DP1) to target receptors by TG11-77.HCl. TG11-77.HCl at 1, 3 and 10 μM concentration tested in human recombinant cell lines (HEK-293 for EP1, 2 and 3; 1321N1 cells for DP1).

**Additional file 11.** **Table S1.** Consumption rate of TG11-77 for mice in cohort 1 and cohort 2. The consumption rate was measured based on the weekly average body weight (g) and volume consumed (ml/day). The final drug concentration was measured based on 92.5% compound recovery of the actual dose from drinking water after 7 days at room temperature. Formula used for daily drug (free base) consumption (mg/kg/day) = ((solution consumed*0.5*1000)/body weight) *0.847). Factor 0.847 was obtained from the ratio of molecular weight between HCl salt and free base of the drug (400/472). We have also looked at consumption rates separately in males and females. Male mice consumed more drinking water than females but it was proportional to their body weights and no differences were found between sexes.

## Data Availability

All data analyzed and presented in this study are available from the corresponding author on reasonable request.

## Abbreviations

AD: Alzheimer’s disease
PGE_2_: Prostaglandin-E_2_
EP2: Prostaglandin-E_2_ receptor-2
PGD_2_: Prostaglandin-D_2_
PGF_2_: Prostaglandin-F_2_
PGI_2_: Prostaglandin-I_2_
TXA_2_: Thromboxane-A_2_
DP1: Prostaglandin-D_2_ receptor-1
IP: Prostaglandin-I_2_ receptor
EP4: Prostaglandin-E_2_ receptor-4
GFAP: Glial fibrillary acidic protein
COX-2: Cyclooxygenase 2
qRT-PCR: Quantitative real time polymerase chain reaction
IL-1β: Interleukin-1β
NFT: Neurofibrillary tangle
TNF: Tumor necrosis factor alpha
CCL2: Chemokine (C–C motif) ligand 2
IL-6: Interleukin 6
GAPDH: Glyceraldehyde 3-phosphate dehydrogenase
HPRT1: Hypoxanthine phosphoribosyl transferase 1
TG11-77.HCl: Potent and selective EP2 receptor antagonist
nTg: Non-transgenic
NMDA: N-methyl-d-aspartate
LPS: Lipopolysaccharide
Aβ: Amyloid-beta
APP: Amyloid precursor protein
BV2-hEP2: Human EP2 expressed BV2 cell line
CBC: Complete blood count
HGB: Hemoglobin
RBC: Red blood cells
WBC: White blood cell
PDWc: Platelet distribution width
HCT: Hematocrit
CSF: Cerebrospinal fluid
5xFAD: Mouse expressing APP and PSEN1 transgenes with a total of five familial AD-mutations
