# Supplementary figures and images for: Prostaglandin EP2 receptor antagonist ameliorates neuroinflammation in a two-hit mouse model of Alzheimer’s disease
Source: J Neuroinflammation. 2021 Nov 20;18:273. doi: 10.1186/s12974-021-02297-7 (PMC8605573; doi:10.1186/s12974-021-02297-7)

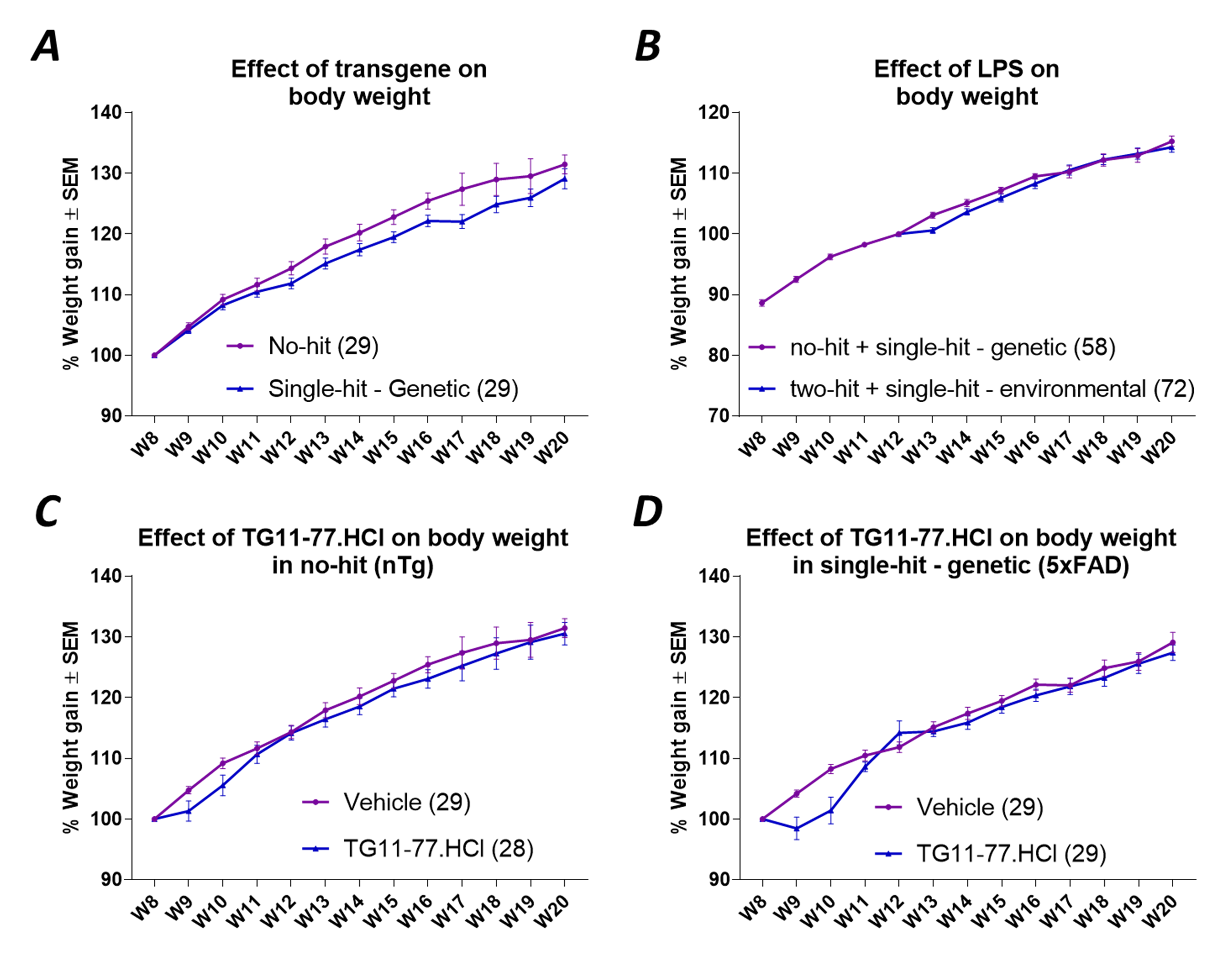

Supplement: Supplementary file 1 — Additional file 1: Fig. S1. No adverse effect of transgene, LPS and TG11-77.HCl on body weight gain in different cohorts of mice. The % body weight gain was measured weekly once from week 8 to week 20 in cohort 1 (no-hit and single-hit—genetic) (A) and from week 12 to week 20 in cohort 2 (single-hit—environmental and two-hit) mice (males and females combined) (B). (C) % weight gain upon TG11-77.HCl treatment in no-hit (C) and single-hit—genetic mice (D). Two-way repeated measure ANOVA with Sidak’s multiple comparisons test was applied. No statistical significance between groups was found on these A–D measures. Data are mean ± SEM. [file 12974_2021_2297_MOESM1_ESM.tif]

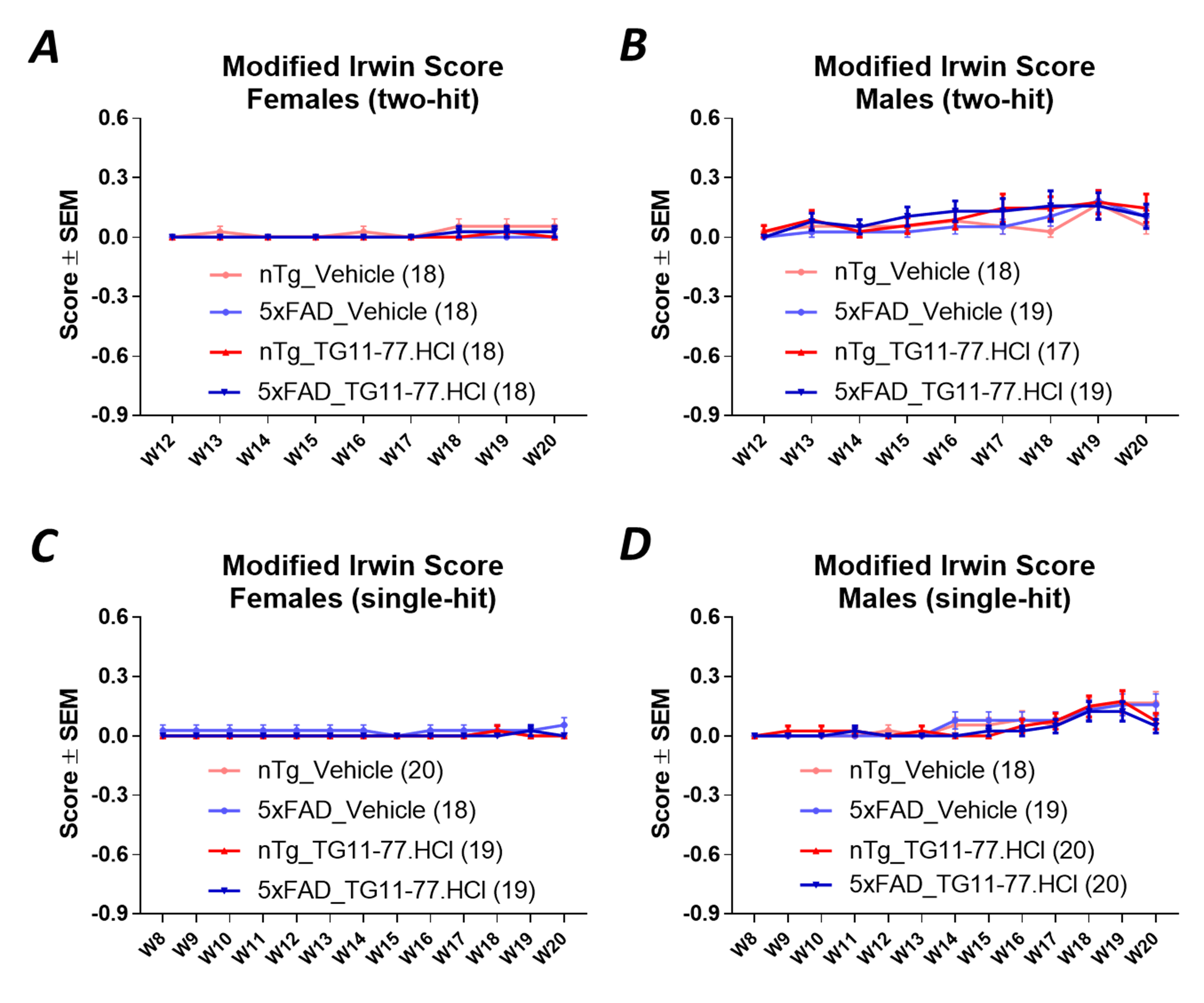

Supplement: Supplementary file 2 — Additional file 2: Fig. S2. No adverse effect of TG11-77.HCl treatment on behavioral and physical activities. Modified Irwin scores showing a cumulative score (range 0–14) from seven behavioral parameters in the mice from both two-hit (A, B) and single-hit cohorts (C, D) treated either with vehicle or TG11-77.HCl. Two-way repeated measure ANOVA with Sidak's multiple comparisons test was applied. No statistical significance between groups was found on these A–D measures. Data are mean ± SEM. [file 12974_2021_2297_MOESM2_ESM.tif]

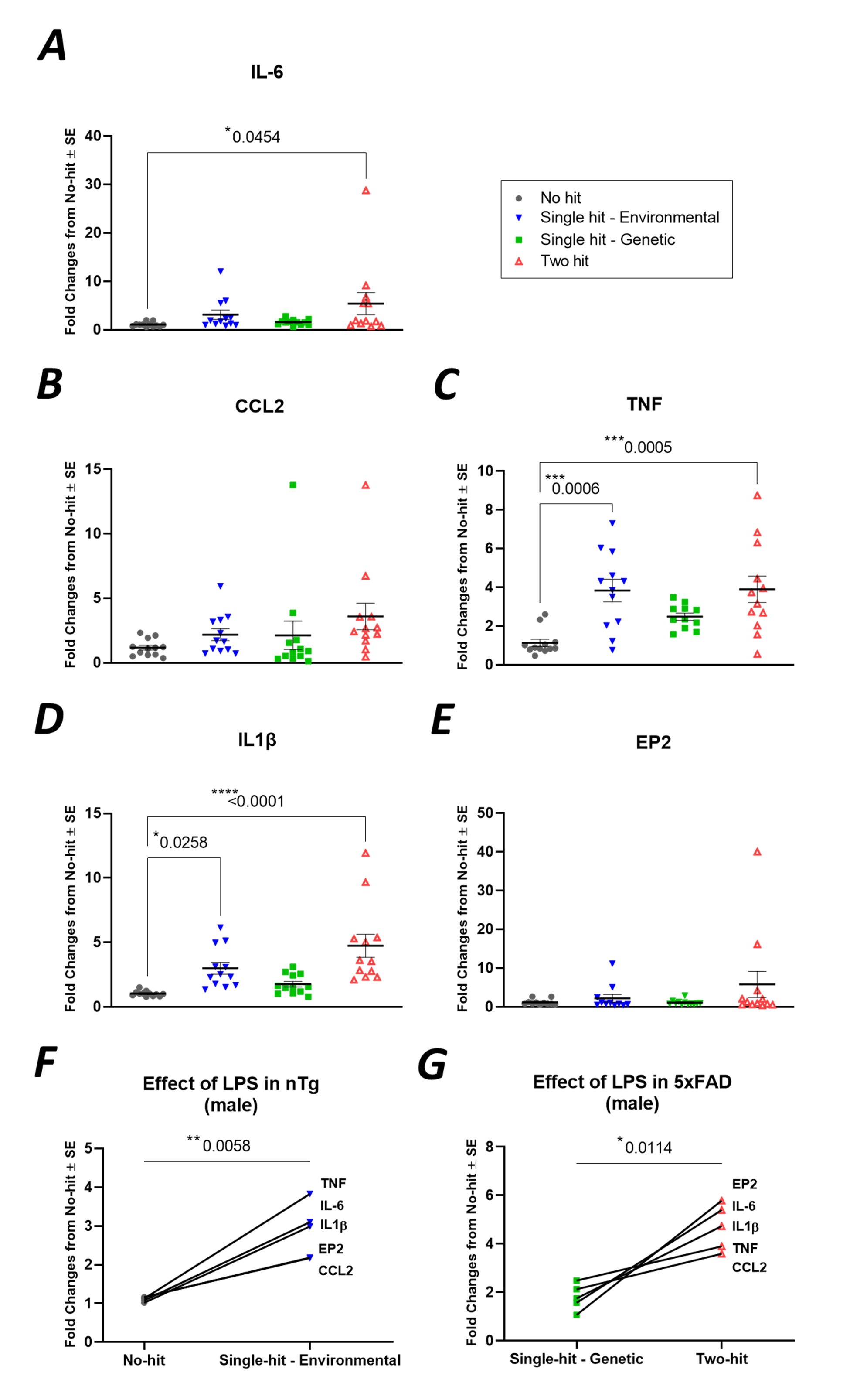

Supplement: Supplementary file 3 — Additional file 3: Fig. S3. LPS induces neuroinflammation in neocortex of two-hit 5xFAD males. (A–E) mRNA fold changes of individual proinflammatory mediators in all hits (environmental and/or genetic) compared to no-hit mice. (F) LPS induced elevation in the group of proinflammatory mediators in single-hit—environmental mice from no-hit mice. (G) LPS induced elevation in the group of proinflammatory mediators in two-hit mice from single-hit—genetic mice. All the groups were normalized to no-hit mice. For individual endpoint analysis one-way ANOVA with Dunnett’s multiple comparisons test was applied (A–E). For group analysis between different hits, paired t test was applied for the series of pro-inflammatory genes (F, G). P values were set to be significant at * = < 0.05, ** = < 0.01, *** = < 0.001 and **** = < 0.0001. Data are mean ± SEM. [file 12974_2021_2297_MOESM3_ESM.tif]

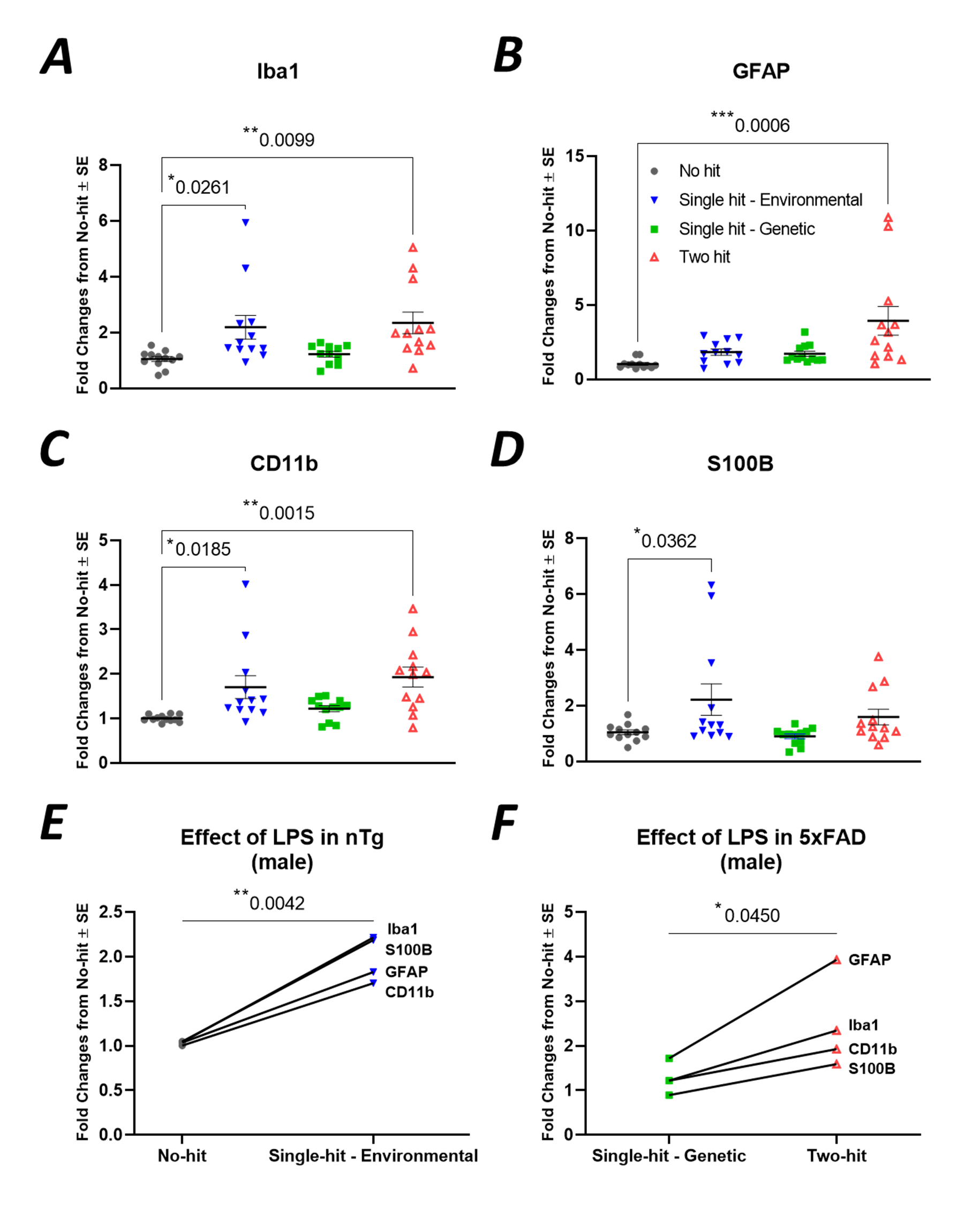

Supplement: Supplementary file 4 — Additional file 4: Fig. S4. LPS induces gliosis in neocortex of two-hit 5xFAD males. (A–D) mRNA fold changes of individual astroglial and microglial markers in all hits (environmental and/or genetic) compared to no-hit mice. (E) LPS induced elevation in the group of glial markers in single-hit—environmental mice from no-hit mice. (F) LPS induced elevation in the group of glial markers in two-hit mice from single-hit—genetic mice. All the groups were normalized to no-hit mice. For individual endpoint analysis one-way ANOVA with Dunnett’s multiple comparisons test was applied (A–E). For group analysis between different hits, paired t test was applied for the series of pro-inflammatory genes (F, G). P values were set to be significant at * ≤ 0.05, ** ≤ 0.01 and *** ≤ 0.001. Data are mean ± SEM. [file 12974_2021_2297_MOESM4_ESM.tif]

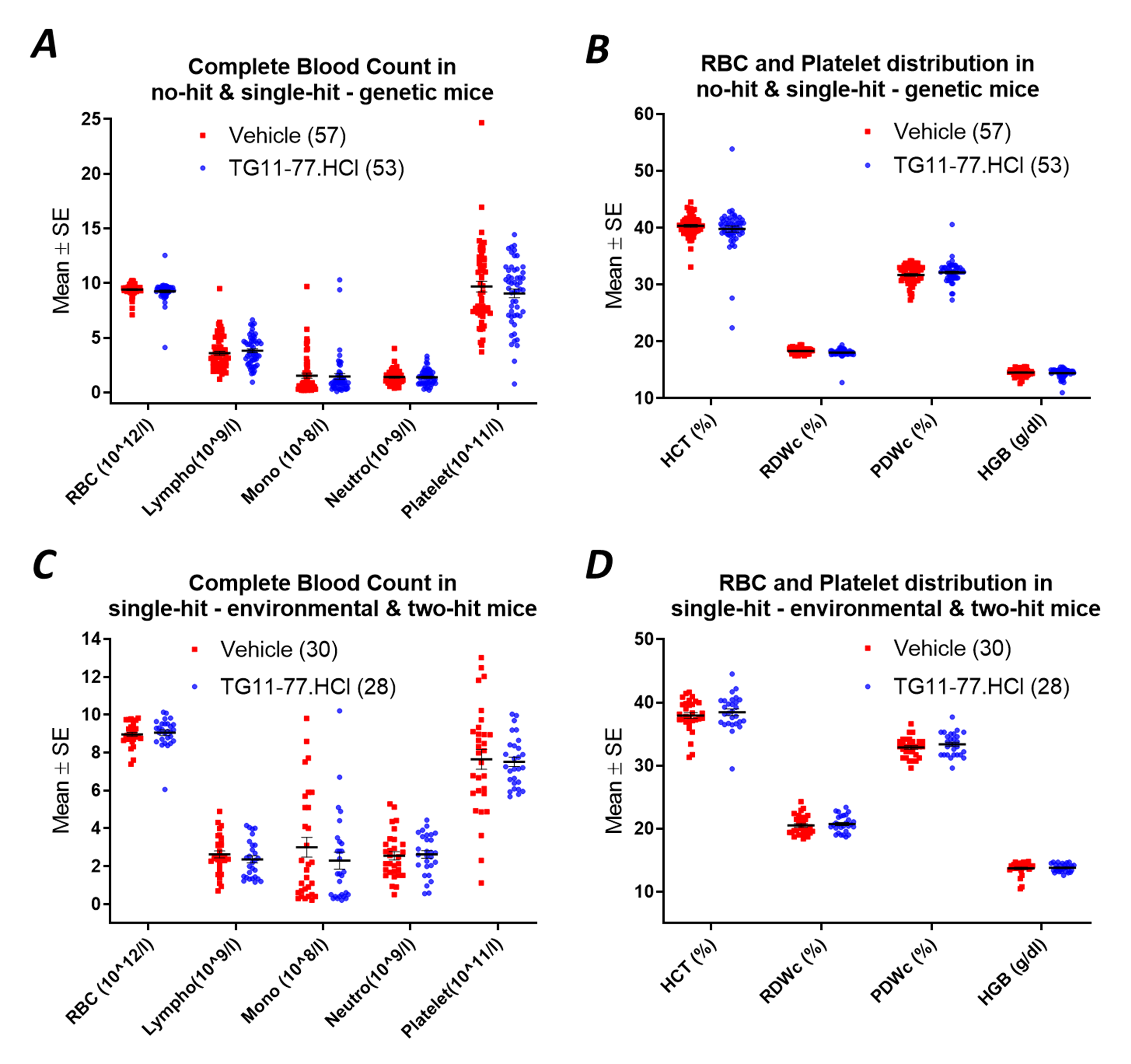

Supplement: Supplementary file 5 — Additional file 5: Fig. S5. EP2 antagonist does not reverse LPS induced anemia of inflammation. (A, B). Complete blood count (CBC) and cell distribution analysis in no-hit and single-hit—genetic combined cohort, upon TG11-77.HCl treatment. (C, D) CBC counts and distribution in single-hit—environmental and two-hit mice combined in presence or absence of TG11-77.HCL treatment. Multiple unpaired t test with Bonferroni correction was applied between groups. P values were set to be significant at * = < 0.05, but no significance between groups was found on these A–D measures. Data are mean ± SEM. [file 12974_2021_2297_MOESM5_ESM.tif]

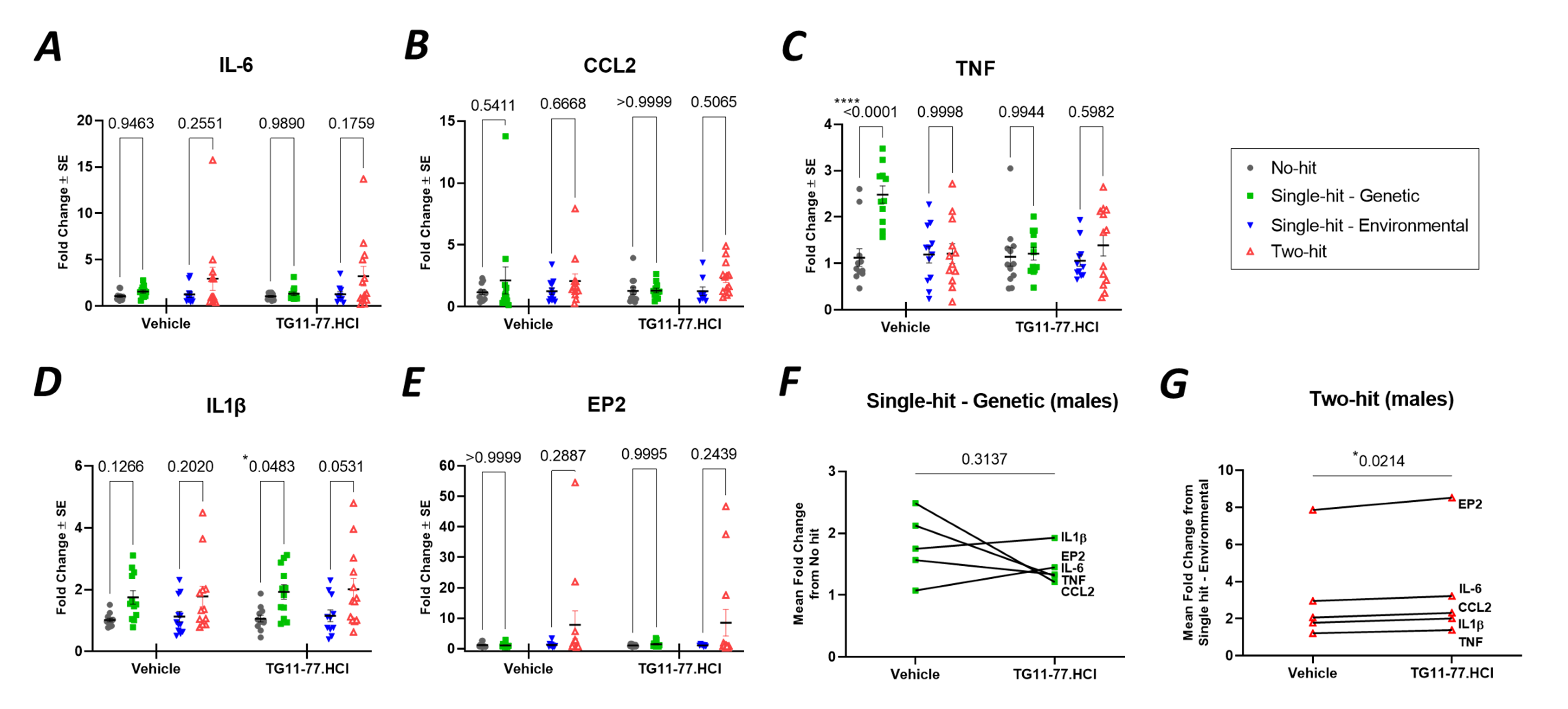

Supplement: Supplementary file 6 — Additional file 6: Fig. S6. No anti-inflammatory effect of EP2 antagonist in single-hit and two-hit 5xFAD males. (A–E) Effect of single-hit (genetic) and two-hit (genetic and environmental) on neuroinflammatory markers in brain neocortex of 5xFAD male mice treated with or without EP2 antagonist TG11-77.HCl. The fold changes in 5xFAD groups (single-hit—genetic or two-hit) were normalized to their respective nTg groups (no-hit or single-hit—environmental). (F) Pairwise effect of TG11-77.HCl treatment in single-hit—genetic males. (G) Pairwise effect of TG11-77.HCl treatment in two-hit males. For individual endpoint analysis two-way ANOVA (hit, treatment) with Tukey's multiple comparisons test was applied (A–E). For group analysis between vehicle and TG11-77.HCl treatment among different hits, paired t-test was applied for the series of pro-inflammatory genes (F, G). P values were set to be significant at * ≤ 0.05 and **** ≤ 0.0001. Data are mean ± SEM. [file 12974_2021_2297_MOESM6_ESM.tif]

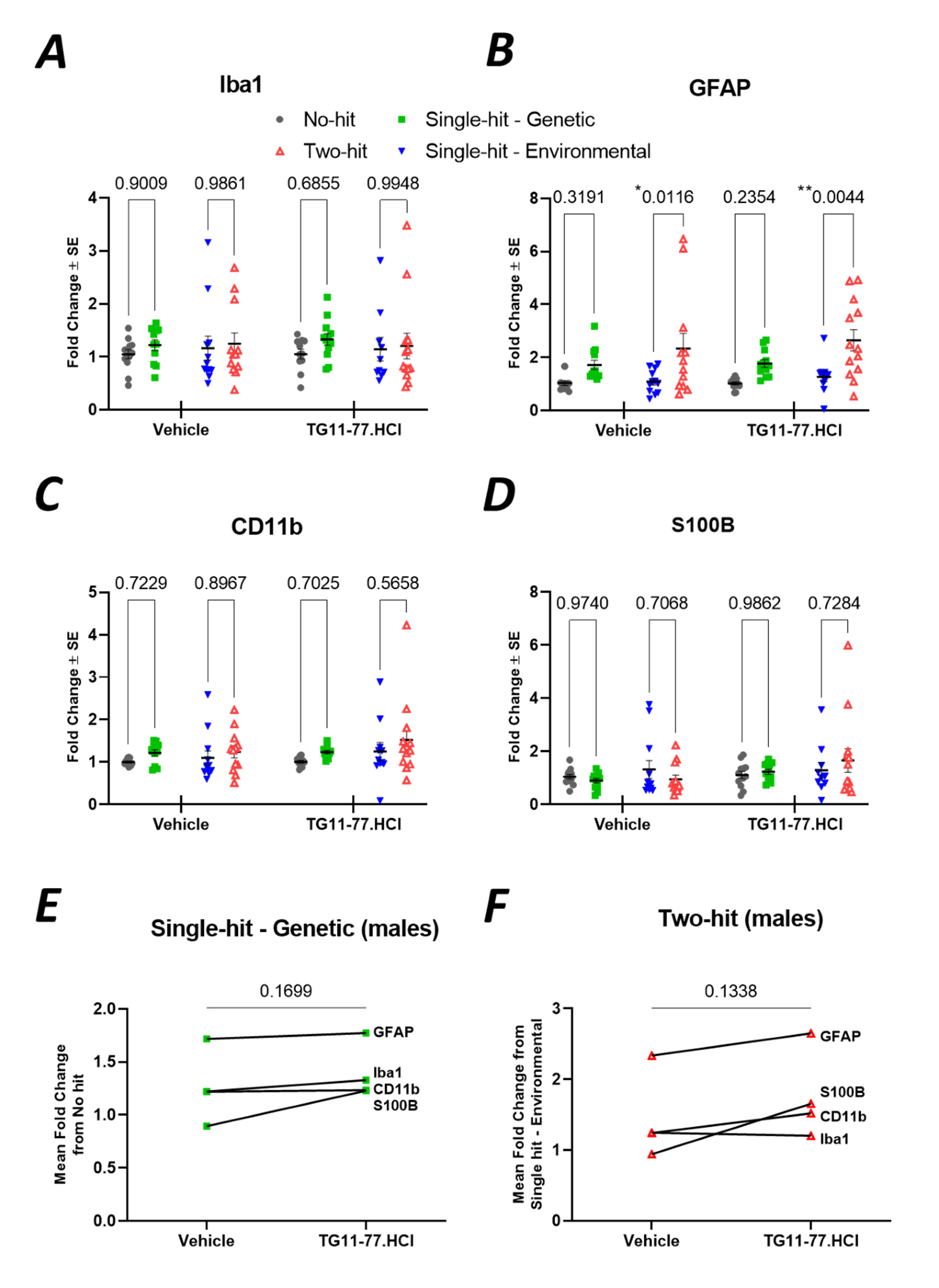

Supplement: Supplementary file 7 — Additional file 7: Fig. S7. No effect of EP2 antagonist on glial markers in single-hit and two-hit 5xFAD males. (A–D) Effect of single-hit (genetic) and two-hit (genetic and environmental) on astroglial and microglial markers in brain neocortex of 5xFAD male mice treated with or without EP2 antagonist TG11-77.HCl. The fold changes in 5xFAD groups (single-hit—genetic or two-hit) were normalized to their respective nTg groups (no-hit or single-hit—environmental). (E) Pairwise effect of TG11-77.HCl treatment in single-hit—genetic males. (F) Pairwise effect of TG11-77.HCl treatment in two-hit males. For individual endpoint analysis two-way ANOVA (hit, treatment) with Tukey’s multiple comparisons test was applied (A–D). For group analysis between vehicle and TG11-77.HCl treatment among different hits, paired t test was applied for the series of glial genes (E, F). P values were set to be significant at * ≤ 0.05 and ** ≤ 0.01. Data are mean ± SEM. [file 12974_2021_2297_MOESM7_ESM.tif]

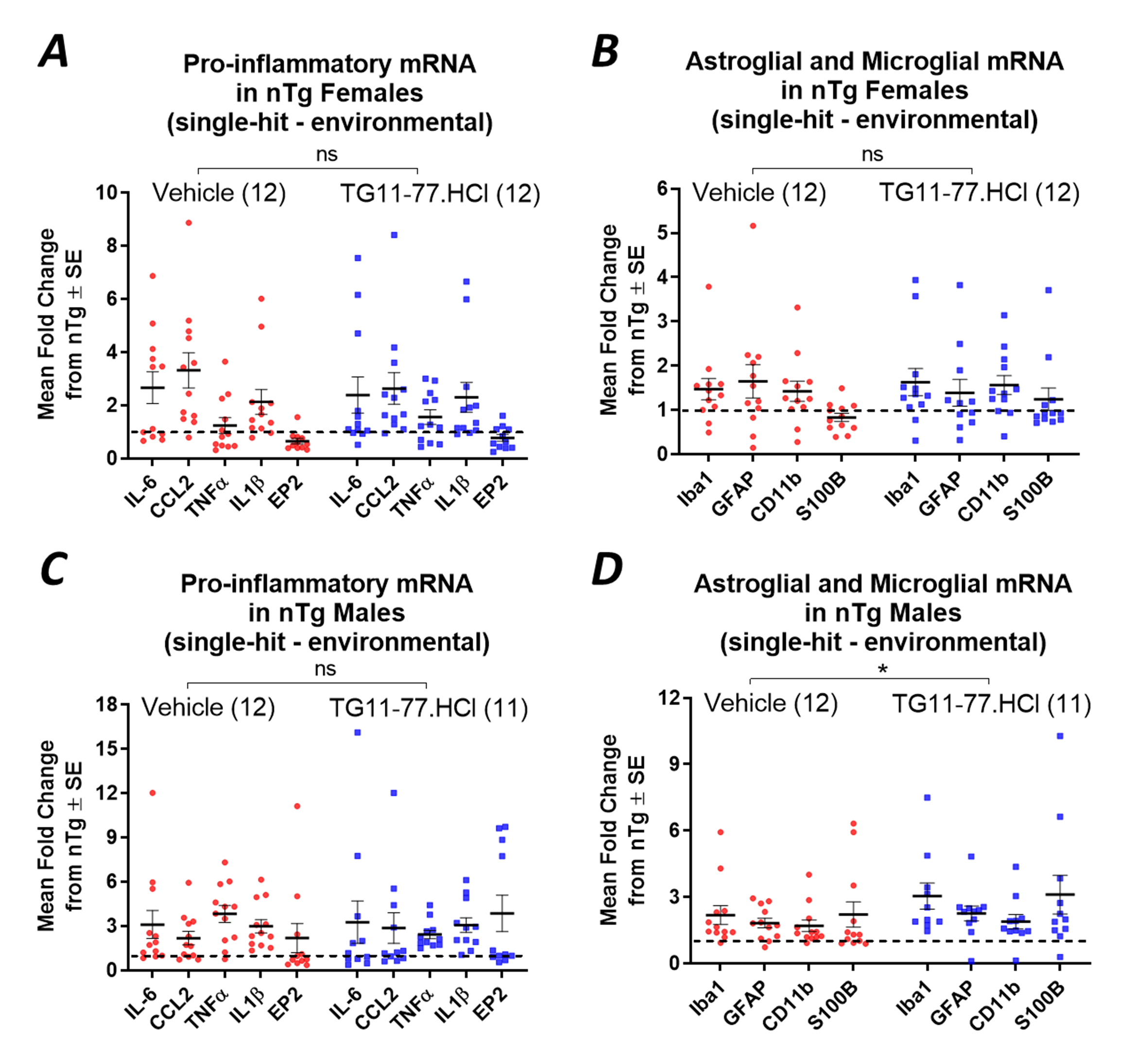

Supplement: Supplementary file 8 — Additional file 8: Fig. S8. No effect of EP2 antagonist on proinflammatory mediators and glial markers in single-hit—environmental nTg mice. Effect of TG11-77.HCL treatment on mRNA level of proinflammatory mediators in single-hit—environmental nTg female (A) and male brain neocortex (C). Effect of TG11-77.HCL treatment on astroglial and microglial markers in single-hit—environmental nTg females (B) and males (D). The fold changes in single-hit—environmental groups were normalized to their respective nTg groups (no-hit), represented by dotted line in the graphs. For group analysis between vehicle and TG11-77.HCl treatment, paired t test was applied. P values were set to be significant at * ≤ 0.05. Data are mean ± SEM. [file 12974_2021_2297_MOESM8_ESM.tif]

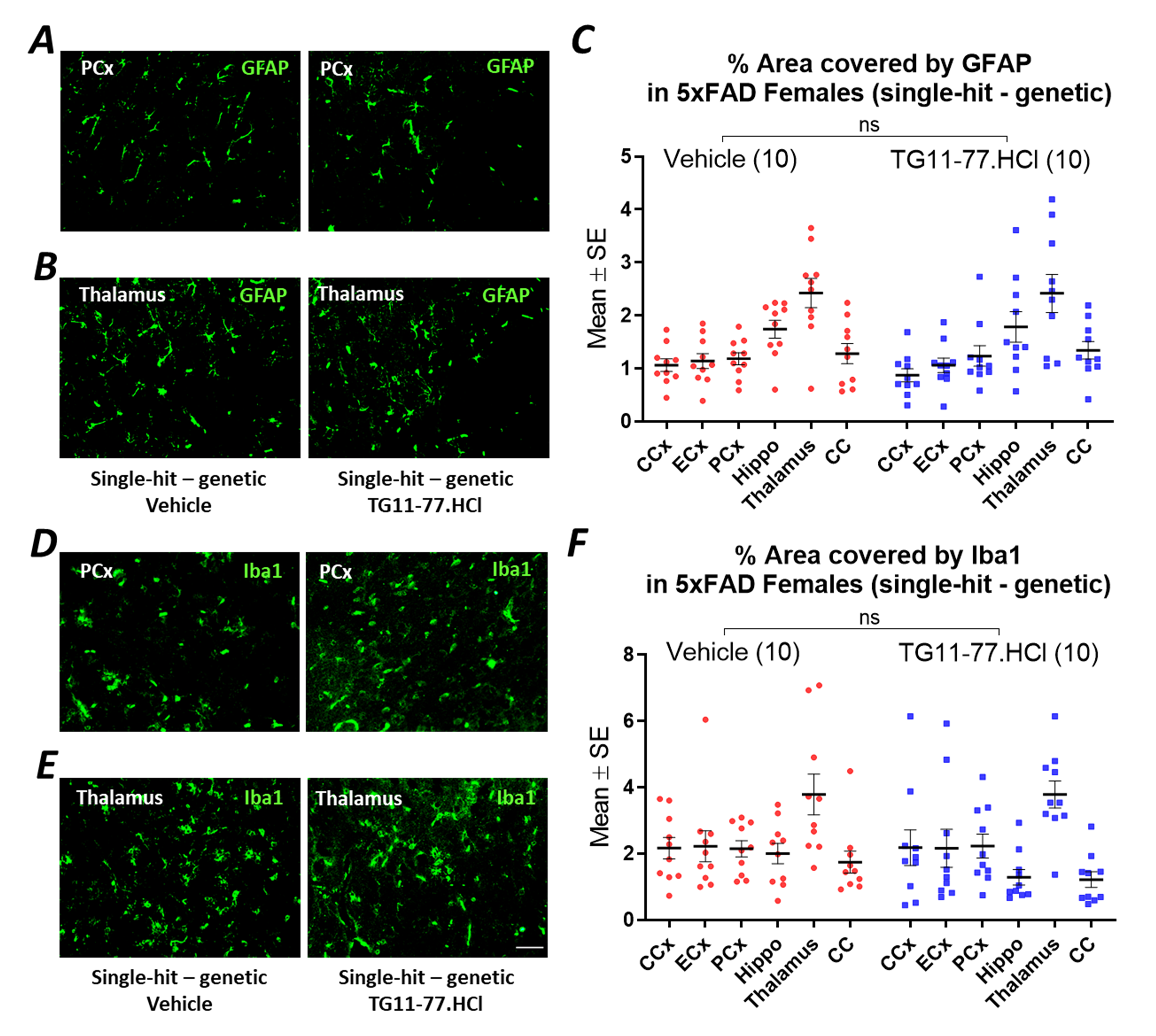

Supplement: Supplementary file 9 — Additional file 9: Fig. S9. TG11-77.HCl did not alter GFAP and Iba1 immunoreactivity in single-hit—genetic 5xFAD female brains. Representative images from TG11-77.HCl or vehicle treated brains showing GFAP immunoreactivity in (A) PCx and (B) thalamus and (C) % area covered in different regions of the brain analyzed by imageJ. Iba1 immunoreactivity in (D) PCx and (E) thalamus and (F) % area covered in different brain regions from single-hit genetic females. Paired t test was applied between groups and multiple unpaired t test with FDR (5%) was applied for multiple comparison. P values were set to be significant at * ≤ 0.05. Scale bar = 50 mm. Data are mean ± SEM. CCx: cerebral cortex; ECx: entorhinal cortex; PCx: piriform cortex; Hippo: hippocampus; CC: corpus callosum. [file 12974_2021_2297_MOESM9_ESM.tif]

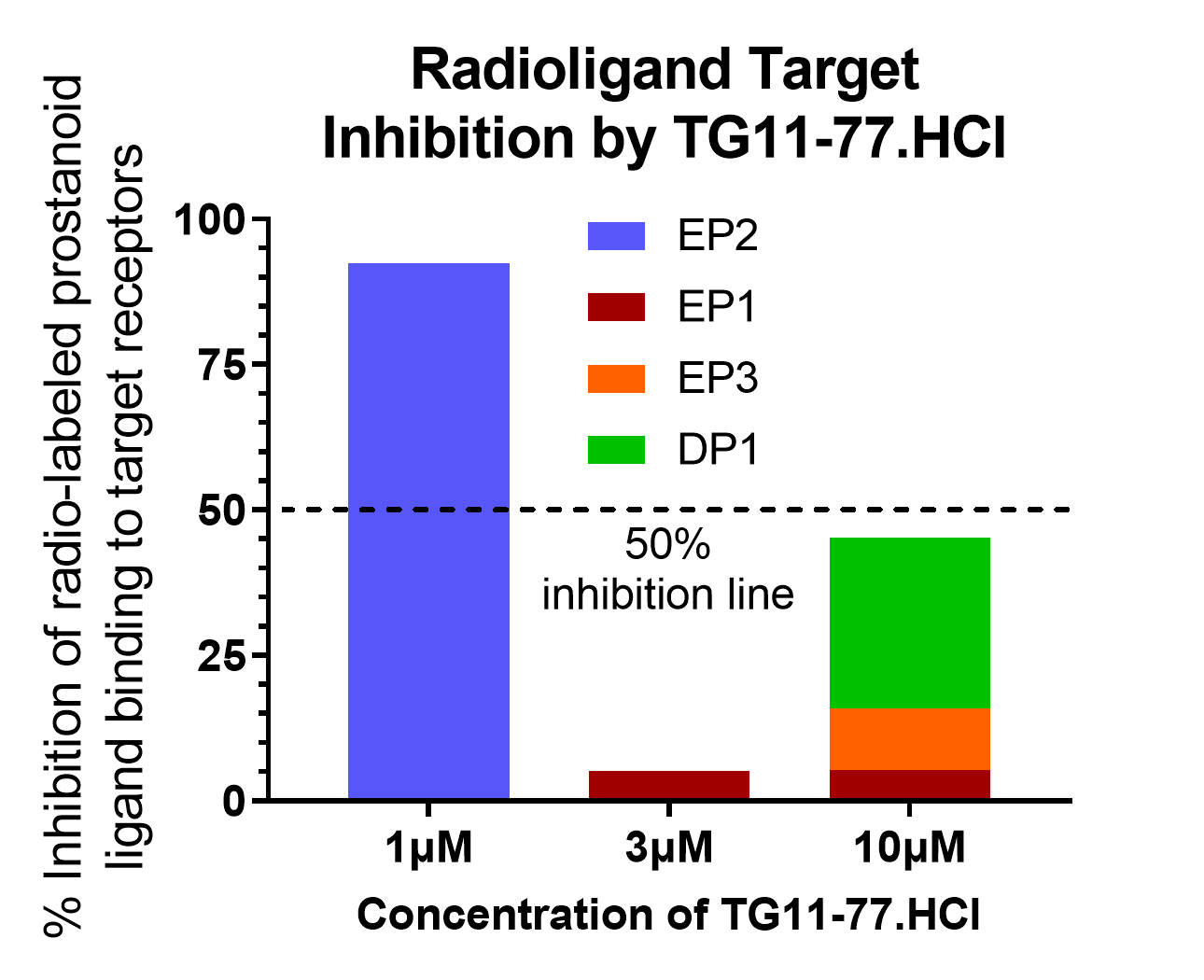

Supplement: Supplementary file 10 — Additional file 10: Fig. S10. TG11-77.HCl exhibits highly selective inhibition of EP2 receptor. Percent inhibition of different radiolabeled prostanoid agonists (PGE2 to EP1–3, or BW245C to DP1) to target receptors by TG11-77.HCl. TG11-77.HCl at 1, 3 and 10 μM concentration tested in human recombinant cell lines (HEK-293 for EP1, 2 and 3; 1321N1 cells for DP1). [file 12974_2021_2297_MOESM10_ESM.tif]
