# Supplementary material for: Prostaglandin EP2 receptor antagonist ameliorates neuroinflammation in a two-hit mouse model of Alzheimer’s disease
Source: J Neuroinflammation. 2021 Nov 20;18:273. doi: 10.1186/s12974-021-02297-7 (PMC8605573; doi:10.1186/s12974-021-02297-7)
Supplement: Supplementary file 11 — Additional file 11. Table S1. Consumption rate of TG11-77 for mice in cohort 1 and cohort 2. The consumption rate was measured based on the weekly average body weight (g) and volume consumed (ml/day). The final drug concentration was measured based on 92.5% compound recovery of the actual dose from drinking water after 7 days at room temperature. Formula used for daily drug (free base) consumption (mg/kg/day) = ((solution consumed*0.5*1000)/body weight) *0.847). Factor 0.847 was obtained from the ratio of molecular weight between HCl salt and free base of the drug (400/472). We have also looked at consumption rates separately in males and females. Male mice consumed more drinking water than females but it was proportional to their body weights and no differences were found between sexes. [file 12974_2021_2297_MOESM11_ESM.docx]

**Table S1:** Consumption rate of TG11-77 for mice in cohort 1 and cohort 2. ^a^

| **TG11-77.HCl** | **Cohort 1 (Single hit)** | | | **Cohort 2 (Two-hit)** | | |
| --- | --- | --- | --- | --- | --- | --- |
| **Average**  **measures for each mouse**  **@ 0.5 mg/ml** | **Body**  **weight**  **(g)** | **Solution**  **drunk**  **(ml/day)** | **Drug (free base) consumption (mg/kg/day)** | **Body**  **weight**  **(g)** | **Solution**  **drunk**  **(ml/day)** | **Drug (free base) consumption (mg/kg/day)** |
| Week 8 | Started | -- | -- |  |  |  |
| Week 9 | 19.68 | 2.96 | 63.63 |  |  |  |
| Week 10 | 20.45 | 3.08 | 63.71 |  |  |  |
| Week 11 | 21.45 | 3.30 | 65.20 |  |  |  |
| Week 12 | 22.35 | 3.43 | 64.92 | Started | -- | -- |
| Week 13 | 22.64 | 3.51 | 65.73 | 22.00 | 3.00 | 57.69 |
| Week 14 | 23.03 | 3.57 | 65.66 | 22.66 | 3.23 | 60.35 |
| Week 15 | 23.58 | 3.61 | 64.86 | 22.88 | 3.24 | 59.90 |
| Week 16 | 23.92 | 3.58 | 63.43 | 23.41 | 3.39 | 61.33 |
| Week 17 | 24.24 | 3.54 | 61.78 | 23.78 | 3.31 | 58.98 |
| Week 18 | 24.46 | 3.50 | 60.54 | 24.08 | 3.37 | 59.24 |
| Week 19 | 27.39 | 3.56 | 55.08 | 24.54 | 3.42 | 58.99 |
| Week 20 | 25.29 | 3.54 | 59.27 | 24.71 | 3.37 | 57.83 |
| Average drug consumption |  |  | 62.82  (mg/kg/day) |  |  | 59.29  (mg/kg/day) |
| **At 92.5% recovery** |  |  | **58.1**  **(mg/kg/day)** |  |  | **54.9**  **(mg/kg/day)** |

**^a^** The consumption rate was measured based on the weekly average body weight (g) and volume consumed (ml/day). The final drug concentration was measured based on 92.5% compound recovery of the actual dose from drinking water after 7 days at room temperature. Formula used for daily drug (free base) consumption (mg/kg/day) = ((solution consumed*0.5*1000)/body weight) *0.847). Factor 0.847 was obtained from the ratio of molecular weight between HCl salt and free base of the drug (400/472). We have also looked at consumption rates separately in males and females. Male mice consumed more drinking water than females but it was proportional to their body weights and no differences were found between sexes.
